# Crystal Structure–Activity Relationship of Some MeO Phenylacrylonitriles: Dual Antimicrobial–Cytotoxic Effects and in Silico Perspectives

**DOI:** 10.1002/open.202500280

**Published:** 2025-06-12

**Authors:** Leyla Babali Özen, Öner Ekici, Furkan Özen, Süleyman Berberler, Gül Özkan, Betül Yılmaz Öztürk, Pınar Oztopcu‐Vatan, Cansu Filik İşçen, Günseli Turgut Cin

**Affiliations:** ^1^ Department of Chemistry Faculty of Science Akdeniz University Pınarbaşı Mahallesi, Dumlupınar Bulvarı, Kampüs, No:578 1952688 Antalya Türkiye; ^2^ Department of Pharmacy Services Durağan Vocational School Sinop University Buzluk Mahallesi, Çiğdem Sokak, No:21 57700 Sinop Türkiye; ^3^ Department of Mathematics and Science Faculty of Education Akdeniz University Dumlupınar Bulvarı, Kampus 07058 Antalya Türkiye; ^4^ Graduate School of Natural and Applied Sciences Eskişehir Osmangazi University Büyükdere, Osmangazi Ünv. No:38 26040 Eskişehir Türkiye; ^5^ Department of Engineering Sciences Faculty of Engineering Izmir Katip Celebi University Balatçık Mahallesi, Havaalanı Şosesi, No:33/2 Balatçık 35620 İzmir Türkiye; ^6^ Central Research Laboratory Application and Research Center Eskişehir Osmangazi University Büyükdere Mahallesi, Prof. Dr. Nabi AVCI Bul., No:4 Eskişehir 26040 Türkiye; ^7^ Department of Biology, Faculty of Science Eskişehir Osmangazi University Büyükdere Mahallesi, Prof. Dr. Nabi AVCI Bulvarı, No:4 26040 Eskişehir Türkiye; ^8^ Department of Mathematics and Science Faculty of Education Eskişehir Osmangazi University Büyükdere Mahallesi, Prof. Dr. Nabi AVCI Bulvarı, No:4 26040 Eskişehir Türkiye

**Keywords:** antimicrobial, crystal structure‐activity, cytotoxic, molecular docking, phenylacrylonitrile

## Abstract

Herein, methoxy‐substituted phenylacrylonitrile derivatives **2(a–c)** are synthesized via Knoevenagel condensation and characterized using fourier‐transform infrared spectroscopy, nuclear magnetic resonance spectroscopy, and X‐ray crystallography (for **2a** and **2b**). Although compounds **2a** and **2b** have previously been reported in terms of their structural features, their dual antimicrobial and anticancer activities, as well as crystallographic structure–activity relationships, have not yet been investigated. Notably, no earlier studies assessed their selective cytotoxicity using both cancerous (MCF‐7) and healthy (L929) cell lines—a gap addressed in this work. Molecular docking analyzes reveal strong binding affinities to biological targets, including penicillin binding protein 2 (PBP2) (−8.4 kcal mol^−1^ for 2c) and CDK1/Cks2 (−9.5 kcal mol^−1^ for 2c), highlighting their dual‐action potential. Antimicrobial assays against nine bacterial strains show minimum inhibitory concentration values ranging from 2.5 to 25 mg mL^−1^, with 2c exhibiting notable activity against gram‐positive bacteria. Cytotoxicity assays demonstrate potent effects against MCF‐7 cells (IC_50_: 34 μM for 2b, 44 μM for **2a**), while **2c** shows broader but moderate activity. The integration of crystallographic, docking, and biological assays underscores the therapeutic potential of these derivatives, with **2(a,b)** emerging as selective candidates for breast cancer treatment.

## Graphical abstract

Methoxy‐phenylacrylonitriles **(2a–c)** are synthesized and characterized by fourier‐transform infrared spectroscopy, nuclear magnetic resonance spectroscopy, and X‐ray crystallography (for **2a,b**). Compound **2b** shows selective cytotoxicity against MCF‐7 cells (IC_50_: 34 μM) and enhances activity against Gram‐positive bacteria. Molecular docking reveals strong binding to CDK1/Cks2 (−9.5 kcal mol^−^^1^), supporting dual therapeutic potential.

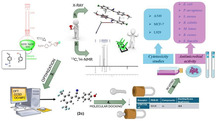

## 1 Introduction

Acrylonitriles play an important role as building blocks in the synthesis of both natural and synthetic molecules with pharmaceutical activity.^[^ ^1^, ^2^ ^]^ The nitrile groups present in the structure of these molecules have attracted increasing attention in recent years, driven by the growing interest in the synthesis of acrylonitrile derivatives and their biological investigation.^[^ ^3^ ^]^ The increase in the number of nitrile‐containing pharmaceuticals has led to the emergence of significant trends regarding the role of nitriles in medicinal agents.^[^ ^3^, ^4^ ^]^

Nitrile groups are one of the functional groups with unique properties in organic chemistry. Due to their short and polarized triple bonds, nitrile groups provide exceptional steric fit and a strong dipole moment,^[^ ^5^ ^]^ playing a critical role in drug design.^[^ ^6^ ^]^ Their small size allows them to interact with biological targets with high affinity, which facilitates their fitting into confined active sites.^[^ ^7^ ^]^ Additionally, nitrile groups interact by forming hydrogen bonds with key amino acid residues located in the active sites of various enzymes.^[^ ^8^ ^]^ Nitrile groups, exhibiting high binding affinity to biological targets, have become indispensable components of modern drug design.^[^ ^9^, ^10^ ^]^ Structurally diverse nitrile‐containing compounds interact with different biological targets, potentially offering effective treatments in a wide range of therapeutic areas. These compounds offer promising therapeutic options for health problems such as cancer, infections, inflammation and metabolic disorders. The nitrile group enhances the biological activity of drugs, allowing for more potent and specific effects. In addition, the development of new derivatives makes it possible to design more effective drugs with fewer side effects. Therefore, nitrile‐containing compounds have significant potential in the field of drug design and discovery.

CA‐4 (combretastatin A‐4), known for its potent anticancer activity and containing four methoxy groups in its structure, stands out in the literature for its antitubulin activity and antivascular properties.^[^ ^11^, ^12^, ^13^ ^]^ The key features of CA‐4 that enhance its interaction with pharmacophore regions include its stilbene backbone with double bonds, aromatic rings, and methoxy groups. Methoxy derivatives are known to have significant effects on biological activities.^[^ ^14^ ^]^ The methoxy group, due to its electron‐donating properties, can increase the electron density in the aromatic ring, thereby strengthening interactions with pharmacophore regions.^[^ ^15^ ^]^ Methoxy‐substituted aryl acrylonitrile compounds are an important subclass in medicinal chemistry and the pharmaceutical industry. Compounds such as CC‐5079^[^ ^16^ ^]^ and TPAT‐AN‐XF^[^ ^17^ ^]^ are notable examples in this field.

Numerous studies have been conducted in the literature on the synthesis of various aryl acrylonitrile derivatives and their biological properties and interactions.^[^ ^18^ ^]^ These studies have comprehensively evaluated the binding affinities of aryl acrylonitrile compounds with biological targets, their pharmacokinetic profiles, and overall efficacy.^[^ ^19^ ^]^ However, detailed data on the properties of aryl acrylonitrile compounds, particularly those with a methoxy group in the para position, synthesized with high yields using these traditional methods, remain limited. The Knoevenagel Condensation is frequently preferred in the synthesis of aryl acrylonitrile derivatives due to its simplicity and effectiveness, providing good yields under mild reaction conditions.^[^ ^20^ ^]^ Current evidence suggests that the synergistic effects of methoxy and nitrile groups may enhance molecular interactions, thereby improving the bioavailability and therapeutic efficacy of these compounds. In this context, the 24‐ and 48 h cytotoxic effects of the obtained methoxy‐substituted aryl acrylonitrile derivatives **2(a–c)** on lung (A549) and breast cancer (MCF‐7) cells, together with their antimicrobial activities, were extensively investigated. In addition, the minimum inhibitory concentration (MIC) values and half maximal inhibitory concentration (IC_50_) of these compounds were determined. The results indicate that these types of compounds have significant potential in both cancer treatment and antimicrobial applications, providing valuable insights for the development of new therapeutic strategies. Among the investigated derivatives, structural analogs of compounds **2a** and **2b** have been previously reported. Specifically, Alam et al.^[^ ^21^ ^]^ described compound **3b**—structurally corresponding to our **2a**—for its anticancer and antimicrobial properties, whereas compound **2b** has primarily been explored for its optical and physicochemical characteristics. The compound has not been studied for any biological activity.^[^ ^22^ ^]^ Notably, these earlier studies did not incorporate healthy cells such as L929, thereby limiting their evaluation of therapeutic selectivity. In contrast, the present study offers a more comprehensive and mechanistically informed assessment by examining the cytotoxic profiles of compounds **2a**–**c** across both cancerous (MCF‐7) and healthy (L929) cell lines, enabling a preliminary estimation of their therapeutic index.

## 2 Experimental and Theoretical Section

### 2.1 Synthesis of Compound 2a

**Scheme 1:**
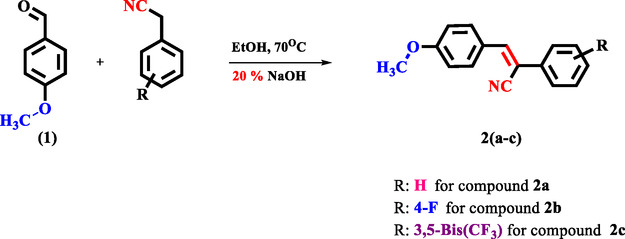
General synthesis scheme of compounds **2**(**a**–**c**).

3‐(4‐methoxyphenyl)‐2‐phenylacrylonitrile (**2a**) was prepared as illustrated in **Scheme** **1**. 4‐methoxybenzaldehyde (3.67 mmol, 0.50 g) and 2‐phenylacetonitrile (3.67 mmol, 0.43 g) were dissolved in ethanol (20 mL). At this temperature, 20% NaOH was gradually added to the reaction mixture until opacity was observed. The reaction was continued with stirring for 30 min.^[^ ^23^, ^24^, ^25^, ^26^ ^]^ The precipitated **2a** compound was filtered after cooling, washed with water, and dried at room temperature. The pale yellow solid substance was obtained as the product. mp: 98 °C. Yield 0.86 g. 92%. *Anal. Calc.* for C15H11NO (MW = 235.29 g moL^−1^): C, 81.43; H, 5.01; N, 6.33. Found: C, 81.40; H, 5.00; N, 6.30%. fourier‐transform infrared spectroscopy (KBr, cm^−1^) 3014*ν*C‐H(Ar.) and 2843*ν*C‐H(aliphatic), 2208*ν*C≡N, 1566, 1602*ν*C*=*C. ^1^H‐nuclear magnetic resonance (NMR) (400 MHz, DMSO‐d6, TMS, ppm): *δ* = 3.84 (3H, s; ‐OCH3), 7.10 (2H, *j* = 8.8 Hz, d; Ar‐H), 7.42 (1H, *j* = 7.2 Hz, t; Ar‐H), 7.50 (2H, *j* = 7.2 Hz, t; Ar‐H), 7.73 (2H, *j* = 7.2 Hz, d; Ar‐H), 7.96 (2H, *j* = 8.8 Hz, d; Ar‐H), 7.98 (1H, s; Ar‐CH=C‐CN). ^13^C‐APT‐NMR (400 *MHz*, DMSO‐d6, TMS, ppm): *δ* = 55.89 C^1^, 161.64 C^2^_(ipso)_, 114.93 C^3^, 131.63 C^4^, 126.71 C^5^, 142.97 C^6^, 107.53 C^7^, 118.90 C^8^, 134.64 C^9^, 125.95 C^10^, 129.260 C^11^, and 129.24 C^12^. (See Supporting Information for compounds **2b** and **2c**).

### 2.2 X‐ray Crystallography

Single‐crystal X‐ray diffraction data for the compounds **2a** and **2b** were obtained using a Rigaku Oxford XCalibur diffractometer, featuring an EOS charge‐coupled device detector and MoKα radiation (*λ* = 0.7107 Å), at ambient temperature. Data acquisition, unit cell refinement, data reduction, and absorption corrections were conducted with the CrysAlisPro software.^[^ ^27^ ^]^ The structure was determined and refined using direct methods, employing SHELXT for structure solution and SHELXL for refinement within the OLEX2 software suite.^[^ ^28^, ^29^, ^30^ ^]^ (See Supporting Information for crystallographic results).

### 2.3 Microorganisms and Antimicrobial Activity

The antibacterial and antifungal activities were reported utilizing the method described by the Clinical & Laboratory Standards Institute.^[^ ^31^, ^32^ ^]^ Agar diffusion method was used to determine the antimicrobial activity of the synthesized substances. The media used were mueller hinton agar (MHA) for bacteria and sabouraud‐dextrose agar (SDA) for fungi (Merck), as recommended by the manufacturer.

### 2.4 Cell Lines and Compounds Preparation

We investigated the cytotoxic effects of acrylonitrile derivatives **2(a–c)** on human lung carcinoma (A549, CCL‐185) cells purchased from American Culture Collection and human breast carcinoma (MCF‐7) cells purchased from Republic of Turkey Ministry of Agriculture and Forestry Şap Institute, (HUKUK, 00092502), In addition, mouse fibroblast cells (L929, CCL‐1) purchased from American Culture Collection were used to observe the effects of the compounds on healthy cells. In 25 T Flask, A549 cells were grown in dulbecco's modified eagle's medium, MCF‐7, and L929 cells were grown in eagle's minimum essential medium (EMEM), 10% fetal bovine serum and 1% Penicillin‐Streptomycin at 37 °C in a 5% CO_2_ and 100% humidified incubator. Cell proliferation was checked every day. Cells were taken into the experiment when the cell density reached 70% to 80%. (See Supporting Information for experimental preparation of biological studies).

### 2.5 Optimization and in Silico Studies

In this stage of the study, GaussView and Gaussian 09 programs were used to prepare the input files of the synthesized compounds, visualize and optimize the molecular structures.^[^ ^33^, ^34^ ^]^

3D structures of all proteins (PDB: 6O0K, 6GU6, 2AZ5, 1MWT) used in docking studies were downloaded from the Protein Data Bank. The minimum binding energy of the interactions between ligand and proteins was found using AutoDockTools (ADT) Version 1.5.7.^[^ ^35^ ^]^ (See Supporting Information for summary of calculated data and coordinates).

## 3 Result and Discussion

### 3.1 Details of Single Crystal X‐ray Crystallography

**Figure 1:**
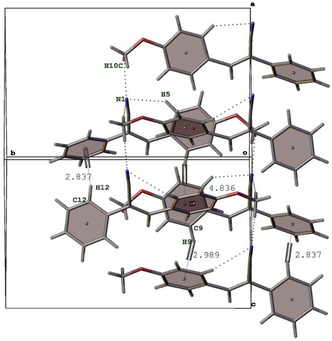
The molecular arrangement of the **2a** along the (101) plane with the noncovalent interactions.

The crystal structure of **2a** is stabilized through a combination of *π*···*π* stacking, C—H···*π* interactions, and weak hydrogen bonds. These interactions play a crucial role in defining the molecular arrangement and can also impact the biological properties of the crystals. The *π*···*π* stacking interactions with perpendicular distance of between 4 Å and 5 Å contribute to the stability of the crystal by facilitating electron density delocalization between adjacent *π*‐systems. The C—H···*π* interactions help align the molecular packing in a (101) plane, influencing the overall stability of the crystal lattice. In addition, short distances such as N1···H10C (2.837 Å) and C9···H9 (2.989 Å) suggest short interactions enhancing molecular stability of the molecule in the solid phase (**Figure** **1**). (See Supporting Information for compound **2b**).

In compound **2a**, the molecular packing is primarily stabilized by *π*–*π* stacking and van der Waals interactions, as confirmed by the X‐ray crystallography data. These interactions lead to a relatively flexible packing arrangement that facilitates intermolecular electron delocalization, potentially enhancing biological membrane permeability. This flexibility in packing may support better adaptability of the molecule within biological targets, thus contributing positively to the observed moderate antimicrobial and cytotoxic activities.

Conversely, in compound **2b**, strong hydrogen bonding interactions dominate the crystal packing due to the presence of electronegative fluorine atoms. Hydrogen bonds form more rigid and directional interactions, resulting in a tighter and more ordered packing structure. While such stabilization improves the solid‐state stability of the compound, it may simultaneously reduce its flexibility in biological environments, potentially limiting optimal fitting within biological receptors. Nevertheless, compound **2b** still exhibits significant cytotoxic activity, particularly against MCF‐7 cells, indicating that strong hydrogen bonding within the molecule also enhances specific target affinity under certain conditions.

### 3.2 Antimicrobial Activity

The synthesized compounds were initially investigated for their antimicrobial activity to elucidate the potential therapeutic utility against various pathogenic microbes using the agar plate diffusion method. The compounds were tested at a concentration of 50 mg mL^−1^, and the antibiotics (ampicillin, kanamycin, and amphotericin B) were used at 5 mg mL^−1^. Antimicrobial activity results of all compounds are presented in **Table** **1**. According to the screening results, compounds **2a**, **2c**, and **2b** exhibited the highest activity against *E. coli*, with inhibition zones of 13, 13, and 12 mm, respectively, compared to 23 mm for ampicillin. Furthermore, compounds **2a**, **2b**, and **2c** showed significant activities against *P. aeruginosa* with growth inhibition values of 13, 12, and 15 mm compared to ampicillin (30 mm). In contrast, compounds **2a**, **2b**, and **2c** were highly active against *S. aureus* with inhibition zones of 14, 13, and 14 mm compared to ampicillin (24 mm). Against *B. subtilis*, compounds **2a**, **2b**, and **2c** demonstrated notable activity, with inhibition zones of 13, 13, and 14 mm, respectively, compared to 32 mm for ampicillin. With respect to *M.luteus*, compounds **2a**, **2b**, and **2c** were most active with inhibition values of 14, 14 and 15 mm compared to ampicillin (44 mm). In the same context, we found that the compounds **2a**, **2b**, and **2c** were the most active against *B. cereus*, with inhibition zones of 15, 13, and 14 mm compared to kanamycin (26 mm). *K.pneumoniae* and *B.cereus* were resistant to ampicillin, while they were susceptible to kanamycin with inhibition values of 30 and 26 mm, respectively. No antimicrobial activity of the compounds was observed against *E.faecalis*, *S.typhimurium, K.pneumoniae*, and for the fungus compared to Amphotericin B (20 mm).

**Table 1:** The in vitro antimicrobial activity of compounds as zone of inhibition (mm).

BACTERIAL STRAINS	2a	2b	2c	Ampicillin	Amphotericin B	Kanamycin
*E.coli*	13 ± 0.71	12 ± 1.41	13 ± 0	25 ± 0.71	–	–
*P.aeruginosa*	13 ± 1.41	12 ± 0.71	15 ± 0.71	30 ± 0.71	–	–
*S.aureus*	14 ± 0.71	13 ± 0	14 ± 1.41	24 ± 1.41	–	–
*B.subtilis*	13 ± 0.71	13 ± 2.12	14 ± 0	32 ± 1.41	–	–
*E.faecalis*	–a)	–	–	34 ± 0.71	–	–
*S.typhimurium*	–	–	–	25 ± 0	–	–
*M.luteus*	14 ± 0.71	14 ± 0.71	15 ± 0.71	44 ± 5.66	–	–
*B.cereus*	15 ± 2.12	13 ± 2.83	14 ± 0.71	–	–	26 ± 1.41
*K.pneumoniae*	–	–	–	–	–	30 ± 0
**FUNGAL STRAIN**	–	–	–	–	–	–
*C.albicans*	–	–	–	–	20 ± 0.71	–
Cons. (mg mL^−1^)	50	50	50	5	5	5

a) “–” indicates no significant inhibitory effect (<6 mm).

The MIC and minimum bactericidal concentration (MBC) were also evaluated for compounds **2(a–c)**, as shown in **Table** **2** and **3**.

**Table 2:** MIC (MIC mg mL^−1^) of compounds 2(a–c).

Strains	2a	2b	2c	Ampicillin	Kanamycin	Amphotericin B
*E.coli*	2.5	25	25	0.03125	–	–
*P.aeruginosa*	5	12.5	12.5	0.0625	–	–
*S.aureus*	12.5	12.5	6.25	0.25	–	–
*B.subtilis*	25	25	25	12.5	–	–
*M.luteus*	6.25	12.5	2.5	0.03125	–	–
*B.cereus*	12.5	12.5	12.5	–	0.03125	–
*E.faecalis*	–	–	–	0.25	–	–
*C.albicans*	–	–	–	–	–	0.25

**Table 3:** MBC (MBC mg mL^−1^) of compounds 2(a–c).

Strains	2a	2b	2c	Ampicillin	Kanamycin	Amphotericin B
*E.coli*	5	–	–	0.03125	–	–
*P.aeruginosa*	5	12.5	12.5	0.0625	–	–
*S.aureus*	25	25	12.5	0.25	–	–
*B.subtilis*	–	–	–	25	–	–
*M.luteus*	6.25	12.5	5	0.03125	–	–
*B.cereus*	12.5	12.5	12.5	–	0.0625	–
*E.faecalis*	–	–	–	0.25	–	–
*C.albicans*	–	–	–	–	–	0.25

As seen in Table 2 and 3, the MIC and MBC values of the compounds vary between 2.5–25 and 5–25 mg mL^−1^, respectively.

The antimicrobial activity of the synthesized acrylonitrile derivatives was evaluated based on MIC and MBC values in comparison with positive controls. For Gram‐negative bacteria, MIC values ranged from 2.5 to 25 mg mL^−1^ for Escherichia coli and 5 to 12.5 mg mL^−1^ for Pseudomonas aeruginosa. In contrast, ampicillin, the positive control, showed much lower MIC values of 0.03125 and 0.0625 mg mL^−1^, respectively. MBC values for the derivatives were also considerably higher than those of ampicillin, indicating limited antibacterial efficacy. Against Gram‐positive bacteria, better activity was observed. MIC values for Staphylococcus aureus ranged between 6.25 and 12.5 mg mL^−1^, while ampicillin showed an MIC of 0.25 mg mL^−1^. MBC values for the derivatives varied from 12.5 to 25 mg mL^−1^. For Bacillus cereus, both MIC and MBC values were 12.5 mg mL^−1^, whereas kanamycin, the control, exhibited superior activity with an MIC of 0.0625 mg mL^−1^. Among the derivatives, compound **2c** showed particularly promising results.

In antifungal assays against Candida albicans, the derivatives showed no detectable activity, with MIC and MBC values significantly higher than those of Amphotericin B.

### 3.3 Cytotoxic Activity

**Figure 2:**
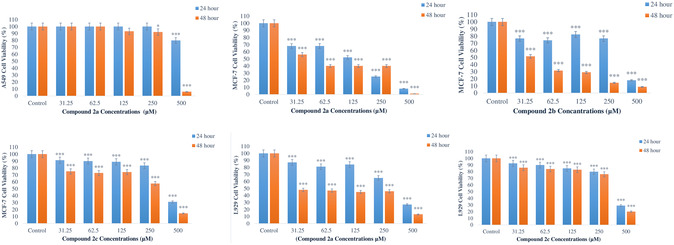
Cytotoxic effects of title compounds on A549, MCF‐7, and L929 cells after 24 and 48 h. The control group was accepted as 100% (*: *p* < 0.05; ***: *p* < 0.001).

The cytotoxic effect of compound **2a** on A549 cells was investigated for 24 and 48 h. As a result of the study, no cell suppressive effect was observed at concentrations of 31.25, 62.5, 125, and 250 μM for 24 h (*p *> 0.05). It was found that the cytotoxic effect started at 500 μM (*p* < 0.001). Cell viability was found to decrease by 20% at this concentration. The IC_50_ value was not determined. As a result of 48 h of treatment, it was observed that the cytotoxic effect started at a concentration of 250 μM (*p* < 0.005) and increased at 500 μM (*p* < 0.001). The decrease in cell viability was determined as 8% for 250 μM and 94% for 48 h. The IC_50_ value was found to be 373 μM for this period (**Figure** **2**).

Compound **2b** applied for 24 and 48 h had no cell suppressive effect on A549 cells at concentrations of 31.25; 62.5; 125; and 250 μM (*p *> 0.005). It was determined that the suppressive effect on cell proliferation for 24 h started at a concentration of 500 μM (*p* < 0.005). It was calculated that cell viability decreased by 9% at this concentration. IC_50_ value could not be calculated. In 48 h of application, it was observed that the suppressive effect on proliferation started at a concentration of 500 μM (*p* < 0.001) and cell viability decreased by 71%. IC_50_ value was calculated as 422 μM (Figure S1, Supporting Information).

In the study, no suppressive effect of compound **2c** on cell proliferation was observed on A549 cells in 24 h, while in 48 h of treatment, it was observed that it was cytotoxic at 500 μM concentration and cell viability decreased by 56% (*p* < 0.001). The IC_50_ value was determined as 473 μM (Figure S2, Supporting Information).

A549 cells were treated with 5‐FU, which was used as positive control in the study, at doses of 31.25; 62.5; 125; 250; and 500 μM for 24 and 48 h. 5‐FU did not show any suppressive effect on A549 cell viability at 24 h in the study (*p *> 0.05). At the end of 48 h of application of 5‐FU to A549 cells, it was determined that the cytotoxic effect started at doses of 250 and 500 μM (*p* < 0.001). It was determined that cell viability decreased by 17% and 77% at these doses, respectively. The IC_50_ value was determined as 386 μM for 48 h (Figure S3, Supporting Information).

Compound **2a** treated with MCF‐7 cells for 24 h was determined to show cytotoxic effect from the first concentration (*p* < 0.001). It was determined that cell viability was reduced by 32, 32, 48, 75, and 92% at 24 h concentration, respectively. IC_50_ value was determined as 131 μM. It was determined that it showed cytotoxic effect on MCF‐7 cells at 48 h as well as 24 h from the first concentration (*p* < 0.001) and the viability in cells was reduced by 44, 60, 60, 60, and 99%, respectively. IC_50_ value was determined as 44 μM (Figure 2).

In the study, it was determined that compound **2b** treated with MCF‐7 cells significantly reduced cell viability at 24 and 48 h (*p* < 0.001). It was determined that the decrease in cell viability was 23, 26, 17, 23, and 82% for 24 h. IC_50_ value was determined as 363 μM for 24 h. In 48 h application, it was determined that cell viability decreased by 49, 68, 71, 86, and 91% respectively, according to the doses. IC_50_ value was determined as 34 μM for 48 h (Figure 2).

The cytotoxic effects of compound **2c** on MCF‐7 cells were investigated, and it was determined that it had a cytotoxic effect at all concentrations at 24 and 48 h (*p* < 0.001). The decrease in cell viability at 24 h was determined to be 9, 10, 11, 17, and 69%, respectively, and 25, 27, 26, 42, and 85%, respectively, at 48 h. The IC_50_ value was determined as 409 μM for 24 h, and 294 μM for 48 h (Figure 2).

In the study, it was determined that 5‐FU suppressed cell viability at all doses in 24 h treatment of MCF‐7 cells (*p* < 0.001). The 5‐FU reduced MCF‐7 cell viability by 23, 23, 34, 50, and 76%, while the IC_50_ value was determined as 250 μM for 24 h. In 48 h treatment, the suppressive effect on cell viability of MCF‐7 cells was observed at all doses (*p* < 0.001) and it was determined that cell viability was reduced by 46, 47, 52, 74, and 95%, respectively. The IC_50_ value was determined as 102 μM for 48 h (Figure S4, Supporting Information).

Compound **2a** was observed to have cytotoxic effects on L929 cells at both 24 and 48 h (*p* < 0.001). At 24 h, cell viability was decreased by 13, 19, 16, 35, and 73%, respectively, and the IC_50_ value was determined to be 351 μM. At 48 h, cell viability was decreased by 52, 53, 55, 54, and 87%, respectively, and the IC_50_ value was determined to be lower than 31.25 μM (Figure 2).

It was determined that the proliferation suppressive effect of compound **2b** on L929 cells was at 24 and 48 h (*p* < 0.001). It was determined that cell viability was reduced by 45, 44, 43, 50, and 75%, respectively, depending on the concentrations in 24 h treatment of L929 cells, and by 52, 54, 53, 53, and 80% in 48 h. IC_50_ value was determined to be lower than 250 μM in 24 h and 31.25 μM in 48 h (Figure S5, Supporting Information).

Similarly, it was determined that the proliferation suppressive effect of compound **2c** on L929 cells was at 24 and 48 h (*p* < 0.001). It was determined that cell viability decreased by 8, 10, 19, 12, and 71%, respectively. IC_50_ value was determined as 410 μM. In the 48 h application, it was determined that it decreased by 14, 16, 20, 24, and 80% and the IC_50_ value was 391 μM (Figure 2).

In the study, it was determined that 24 h treatment of L929 cells with 5‐FU had a cytotoxic effect on the cells (*p* < 0.001) and cell viability was determined to be reduced by 45, 45, 51, 62, and 93%, respectively. In the 48 h treatment (Figure S6, Supporting Information), it was determined that cell viability was reduced by 50, 64, 68, 80, and 93%, respectively (*p* < 0.001). Half maximal inhibitory concentration (IC_50_) results of all compounds are presented in **Table** **4**.

**Table 4:** Cytotoxic activity expressed as IC_50_ values (in μM) of the tested compounds 2(a–c) and 5‐FU against two cancer and a healthy cell lines for 24 and 48 h.

Compounds	Cell lines					
	A549		MCF‐7		L929	
	24 h	48 h	24 h	48 h	24 h	48 h
2a	>500	373	131	44	351	<31.25
2b	>500	422	363	34	250	<31.25
2c	>500	473	409	294	410	391
5‐FUa)	>500	386	250	102	109	33

a) 5‐Fluorouracil.

### 3.4 Molecular Docking Studies

**Figure 3:**
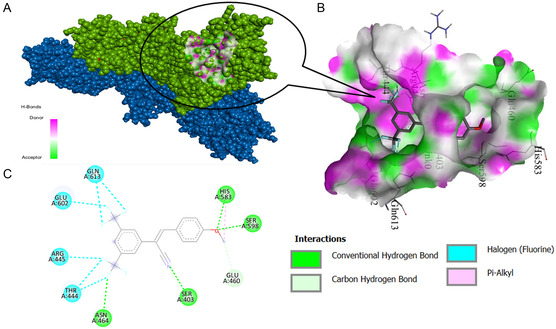
A) Binding pose profile, B) 3D, and C) 2D representation of 1MWT‐ligand **2c** interaction with large binding energy as a result of molecular docking.

In this study, the relationship between the biological activities of the synthesized methoxy‐substituted phenylacrlonitrile derivatives and molecular docking analysis were also evaluated. The theoretical findings of antimicrobial and cytotoxic studies obtained in the light of experimental data and calculations can be summarized as follows:

PBP2 is a protein with transpeptidase activity that plays a critical role in bacterial cell wall biosynthesis. This protein is involved in the cross‐linking process of the bacterial cell wall and is therefore important as an antibiotic target. Inhibition of PBP2 inhibits the synthesis of the bacterial cell wall, triggering bacterial death. The binding affinities of the compounds with PBP2 were examined through docking analysis, and the binding energies are presented in **Table** **5**. The highest binding affinity was shown by compound **2c** (−8.4 kcal moL^−1^). The docking score of this compound partially supports that it is one of the most effective antimicrobial compounds experimentally. Compared to penicillin (−7.7 kcal moL^−1^), the binding capacity of compounds **2a** and **2b** to the target protein is close. Hydrogen bond location and docked poses of ligand **2c**–protein pair were achieved and are illustrated in **Figure** **3** (Figure S7–S9, Supporting Information).

**Table 5:** Docking scores and residues of compounds for PBP2.

Receptor	PDB ID	Compounds	Docking Score [kcal moL^−1^]	Residues (Interactions with Ligand)
PBP2	1MWT	2a	−7.4	HIS A:583, SER A:598, GLU A:460, MET A:641, ALA A:642, SER A:403
PBP2	1MWT	2b	−7.6	HIS A:583, SER A:598, GLU A:460, ALA A:642, SER A:403, GLU A:602
PBP2	1MWT	2c	−8.4	HIS A:583, SER A:598, GLU A:460, SER A:403, ASN A:464, THR A:444, ARG A:445, GLU A:602, GLN A:613
PBP2	1MWT	Penicillina)	−7.7	LYS A:406, SER A:403, ALA A:642, TYR A:446, ASN A:464, GLU A:602, GLN A:521

a) RMSD: 1.986.

A significant correlation was observed between the obtained docking scores and the experimental antimicrobial activity values. The compound **2c** showed both the best docking score and low MIC values against Gram‐positive bacteria. However, it should be noted that docking scores are not the only determinant of antimicrobial activity, and factors such as biological membrane permeability and metabolic stability may also play a role in the activity.

The integration of experimental cytotoxicity data and theoretical molecular docking results provides a comprehensive understanding of the potential anticancer activity of the synthesized methoxy‐substituted phenylacrylonitrile derivatives **2(a–c**). The docking scores suggest a theoretical potential for anticancer activity, particularly in compound **2c**. However, the correlation with in vitro cytotoxicity data was only partial. While docking predicted strong affinities for several targets, the experimental results revealed that compounds **2a** and **2b** showed greater cytotoxicity on MCF‐7 cells than compound **2c**. These findings emphasize that molecular docking offers valuable insights, but actual biological efficacy depends on additional factors such as cellular uptake, metabolic stability, and target accessibility. For CDK1/Cks2, the docking scores and protein‐ligand interactions of compounds **2(a–c)** are listed in **Table** **6** (Table S1 and S2, Supporting Information). Molecular docking studies revealed that compound **2c** exhibited the highest binding affinity among the tested derivatives, with significant interactions observed for key oncogenic proteins such as CDK1/Cks2 (−9.5 kcal moL^−1^), TNF‐alpha (−9.0 kcal moL^−1^), and BCL‐2 (−8.7 kcal moL^−1^). These docking scores suggest strong molecular interactions with cancer‐related targets, indicating a high potential for inhibitory activity.

**Table 6:** Docking scores and residues of compounds for CDK1/Cks2.

Receptor	PDB ID	Compounds	Docking Score [kcal moL^−1^]	Residues (Interactions with Ligand)
CDK1/Cks2	6GU6	2a	−7.5	ALA A:145, ALA A:31, PHE A:80, LEU A:135, VAL A:64, ILE A:10, LYS A:89
CDK1/Cks2	6GU6	2b	−7.8	LYS A:89, ILE A:10, ALA A:145, VAL A:18, ALA A:31, PHE A:80, VAL A:64, LEU A:135
CDK1/Cks2	6GU6	2c	−9.5	LYS A:89, ILE A:10, VAL A:18, ALA A:31, PHE A:80, GLU A:81, LYS A:33, ASP A:146, ASN A:133, GLN A:132, LEU A:135, ALA A:145
CDK1/Cks2	6GU6	Dinacicliba)	−9.1	ILE A:10, LEU A:83, LEU A:135, GLU A:81, PHE A:82, ALA A:31, PHE A:80, VAL A:18, VAL A:64, ALA A:145, LYS A:33, ASN A:133
CDK1/Cks2	6GU6	5‐FU	−4.8	LEU A:83, LEU A:135, ILE A:10

a) RMSD: 1.122.

This divergence between theoretical and experimental results highlights the complexity of predicting in vitro behavior solely based on docking simulations. The observed discrepancies reinforce the need to interpret docking scores as one of several parameters in early‐stage drug discovery rather than as definitive predictors of biological potency. While compound **2c** exhibited the highest docking scores, its cytotoxic activity in MCF‐7 and A549 cell lines was moderate, with IC_50_ values of 294 and 473 μM at 48 h, respectively. Interestingly, compounds **2a** and **2b** showed lower docking scores yet exhibited stronger cytotoxic effects in MCF‐7 cells, with IC_50_ values of 44 and 34 μM, respectively. Although minor discrepancies exist between theoretical and experimental results, our comprehensive analysis (as presented in the Conclusion section) confirms that these variations remain within acceptable limits and ultimately enhance the study's contributions. The IC_50_ value was found to be 373 μM for this period (Figure 2).

An essential aspect of evaluating novel anticancer compounds is their selectivity toward cancer cells over normal cells. The therapeutic index, calculated as the ratio of IC_50_ values in healthy (L929) cells versus cancer cells, revealed that compound **2c** exhibited a safer profile compared to **2a** and **2b**. Specifically, its IC_50_ value in L929 cells was 391 μM, indicating lower toxicity toward healthy cells. Conversely, compounds **2a** and **2b** demonstrated higher cytotoxicity in MCF‐7 cells but also showed considerable toxicity toward L929 cells, necessitating further structural modifications to enhance selectivity.

The crystal structure–efficacy relationship is presented in the conclusion of the study; however, to better contextualize the partial discrepancy between molecular docking predictions and in vitro biological outcomes, potential alternative mechanisms and influencing factors should also be considered:

Although this study did not primarily focus on elucidating the exact mechanisms underlying the observed antibacterial and anticancer activities, various potential pathways could be considered. Molecular docking results revealed notable interactions between the synthesized compounds and key biological targets, including PBP2, CDK1/Cks2, TNF‐α, and BCL‐2. However, these theoretical findings only partially aligned with the in vitro cytotoxicity results. Notably, compound **2c** demonstrated the highest docking affinities, yet compounds **2a** and **2b** exhibited stronger cytotoxic activity on MCF‐7 cells. This discrepancy suggests that docking scores alone may not be sufficient to predict biological efficacy and that other factors—such as cellular uptake, membrane permeability, metabolic transformation, and intracellular target accessibility—could significantly influence the actual biological outcomes.

Furthermore, alternative mechanisms of action may include inhibition of DNA gyrase or topoisomerases, disruption of membrane integrity, induction of oxidative stress via reactive oxygen species, modulation of apoptotic signaling (e.g., BCL‐2 regulation), and interference with epigenetic regulators such as histone deacetylases. These proposed pathways are plausible considering the structural features of the molecules, particularly the electron‐donating methoxy group and the polar nitrile moiety. Moreover, supramolecular interactions observed in X‐ray crystallographic data—such as *π*···*π* stacking and C—H···*π* interactions—may also support molecular stability and target engagement. Taken together, these findings underscore the need for further mechanistic investigations to better understand the structure–activity relationship and to validate the biological relevance of the theoretical predictions.

## 4 Conclusion

In general, the antimicrobial activity of the acrylonitrile derivatives synthesized in the study was better against Gram‐positive bacteria but lower against Gram‐negative bacteria and fungi. Comparisons with positive controls show that the activity level of the synthesized derivatives still needs to be improved. In particular, these derivatives should be optimized by structural modifications to increase the activity against Gram‐negative bacteria and fungi. Future studies should focus on investigating the biological activity mechanism of these derivatives in more detail and obtaining a broader antimicrobial spectrum by modifying their side groups.

In previous studies, the dose‐ and time‐dependent cytotoxic effects of synthesized different acrylonitrile derivatives were investigated in various cell lines.^[^ ^36^ ^]^ In the study, when title derivatives **2(a–c)** were tested on A549 cells for 24 h, no proliferation suppressive effects were observed. Alam et al.^[^ ^21^ ^]^ described compound **3b**‐structurally corresponding to our **2a**‐for its four different cancer cells. In this study, they found the IC_50_ value as 25.51 mg mL^−1^ at 48 h in A549 cells. Compound **2a** did not show any cytotoxic activity in A549 cells within 24 h. At 48 h, the IC_50_ value was found to be 373 μM for 48 h (Figure 2). When our study is compared with this study, may explain the differences in results due to variations in concentration, the number of cells used in cell cultivation, and cytotoxicity methods.

However, in MCF‐7 cells, it was determined that all compounds showed proliferation suppressive effect, and **2a** was the most effective. In 48 h, it was determined that the cytotoxic effects of the compounds increased depending on the dose. The highest proliferation suppressive effect was observed in compound **2b** in MCF‐7 cells (IC_50_ value 34 μM). Unfortunately, compound **2b** also exhibited toxicity toward healthy cells in a dose‐ and time‐dependent manner.

According to the study,^[^ ^37^ ^]^ the cytotoxic effects of synthesized methoxy group‐bearing 47 acrylonitrile derivatives were investigated across 8 different cell lines. The study revealed that compound number 27 suppressed proliferation, particularly in pancreatic adenocarcinoma cells (IC_50_ values ranging from 1.2 to 5.3 μM), indicating that the chemical composition of these derivatives plays a key role in their cytotoxic effects.

In addition, in order to compare the cell proliferation suppressive effect of synthesized compounds, 5‐FU, which is currently used in cancer treatment, was tested on cells at the same concentrations and durations. 5‐FU showed higher dose and time‐dependent toxicity in healthy cells compared to the compounds used in the study. In addition, it was observed that compounds **2a** and **2b** suppressed cell viability more than 5‐FU in MCF‐7 cells. When the results obtained are examined, it is thought that compounds **2a** and **2b** have the potential to be developed as drugs, especially in the treatment of breast cancer.

Compound **2c** showed moderate cytotoxic activity in both MCF‐7 and A549 cells. This level of potency increases the potential of this compound for use in a wide range of cancer types.

The correlation between X‐ray crystallography and molecular docking is critical for validating the structural stability and biological relevance of the studied compounds. The strong hydrogen bonds, short‐range interactions, and dimer formation observed in the structure, which contribute to its stabilization and serve as direct indicators of molecular stability, also play a role in fundamental interactions such as hydrogen bonding, *π*–*π* stacking, and hydrophobic contacts, as shown in Figure S7–S9, Supporting Information, as well as in the binding energy of the compounds to the target molecule.

According to **Table** **7**, X‐ray analysis reveals the absence of classical hydrogen bonding in compound **2a**, whereas no such absence is observed for compound **2b**. This indicates that the crystal packing of compound **2a** is primarily stabilized by *π*–*π* stacking and van der Waals interactions. In contrast, the packing system of compound **2b** is dominated by strong hydrogen bonds due to the presence of fluorine atoms in its structure. The strong hydrogen bonds formed between the ligands enhance the structural stability. This increased stability can influence the binding energy between the ligand and the target protein in two different ways. The expected outcome is a high cytotoxic effect, as it reinforces the interaction and compatibility between the ligand‐protein complex. However, as shown in Table 4, although this expected effect is partially observed for compounds **2b** and **2a** in the MCF‐7 cell line after 48 h of treatment, compound **2c**, which possesses a large number of strong hydrogen bonds, exhibits a low cytotoxic effect. This discrepancy may be due to alternative factors, suggesting the following explanation.

**Table 7:** Details of noncovalent interactions for molecules (Å).

Nonclassical Hydrogen Bonds						
Molecule	D—H···A	D—H	H···A	D···A	D—H···A	Symmetry code
**2a**	**Nonclassical Hydrogen Bonds were not observed.**				–	–
–	C3—H3···N1a)	0.82	2.04	2.864(4)	177	1 − *x*,1/2 + *y*,1/2 − *z*
**2b**	–	–	–	–	–	–
–	C13—H13···F2	0.93	2.55	3.372(4)	148	2 − *x*,1 − *y*,2‐*z*

C—H···*π* Interactions						
–	C—H···Cg	H···Cg	C—H···Cg	C···Cg	C—H,pi	Symmetry code
**2a**	C9**—**H9···Cg3	2.96(8)	134.3	3.668(7)	54	− 1/2 + *x*,1/2 − *y*,*z*
	C15**—**H15···Cg3	2.86(8)	138.3	3.617(6)	55	1/2 − *x*,1/2 + *y*,1/2 + *z*
–	C12**—**H12···Cg4	2.84(6)	133.6	3.543(6)	51	*x*,*y*,*z*
**2b**	C9**—**H9···Cg2	2.85	133	3.55(6)	52	2 − *x*,1 − *y*,1 − *z*
	C15**—**H15···Cg1	2.85	141	3.625(6)	48	1 − *x*,1 − *y*,1 − *z*

a) **Ring's names**: Cg1: C4/C9; Cg2: C10/C15; Cg3: C4/C19; Cg4: C11/C16.

An increase in the number of strong hydrogen bonds within the dimer structure reduces the flexibility of the ligand within the target protein. As this flexibility decreases, the probability of forming a stable binding pocket within the protein also diminishes. While docking scores and binding interactions predict a high cytotoxic effect for compound **2c**, findings from cancer studies indicate otherwise.

## Conflict of Interest

The authors declare no conflict of interest.

## Supporting information

Supplementary Material

## Data Availability

The data that support the findings of this study are available from the corresponding author upon reasonable request.
