# Supplementary material for: Crystal Structure–Activity Relationship of Some MeO Phenylacrylonitriles: Dual Antimicrobial–Cytotoxic Effects and in Silico Perspectives
Source: ChemistryOpen. 2025 Jun 12;14(11):e202500280. doi: 10.1002/open.202500280 (PMC12598805; doi:10.1002/open.202500280)
Supplement: Supplementary file 1 — Supplementary Material [file OPEN-14-e202500280-s001.zip › open202500280-sup-0001-suppdata-S1.pdf]

Supporting Information

This file includes:

1 Experimental……………………………………………………………………………………….S2

2 X-ray Crystallography……………………………………………………………………………...S3

3 Microorganisms and Antimicrobial Activity…………………………………………………….S4

4 Optimization and In Silico Studies……………………………………………………………..S6

5 Details of Single Crystal X-ray Crystallography………………………………………………S10

6 Figures S1-S9…………………………………………………………………………………….S14

7 References………………………………………………………………………………………..S18

1. Experimental

1.1. Chemicals and Equipments

All starting materials and solvents required for the synthesis were sourced from Fluka and Aldrich companies. Melting points were determined using an electrothermal melting point apparatus. FT-IR spectra were recorded on a Bruker Tensor 27 FT-IR spectrometer with a polystyrene film-calibrated KBr disc. Nuclear magnetic resonance (^{1}H and ^{13}C-APT) spectra were obtained using a BRUKER Spectrospin Avance DPX400 Ultrashield (400 MHz) spectrometer, with tetramethylsilane (TMS) as the internal standard.

1.2. Synthesis of Compound 2b

4-methoxybenzaldehyde (3.67 mmol, 0.50 g) and 4-flourophenylacetonitrile (3.67 mmol, 0.49 g) were reacted. The pale yellow solid substance was obtained as the product. Mp: 75℃. Yield: 0.68 g. 87%. *Anal*. Calc. for $C_{16}H_{12}FNO$ (MW= 253.28 g.moL^{-1}): C, 75.88; H, 4.21; N, 5.53. Found: C, 75.28; H, 4.19; N, 5.84. %. FT-IR (KBr, cm-1) 3059 and 3024*v*C-H(Ar.), 2208*v*C≡N, 1514, 1564 and 1593*vC=C and* 822*v*C-F. ^{1}H-NMR (400 MHz, $CDCl_3$, TMS, ppm): δ= 3.90 (s, 3H), 7.01 (d, j=8.8 Hz, 2H), 7.90 (d, j=8.4 Hz, 2H), 7.65 (dd, j=9.2, Hz, j=8.8 Hz, 2H), 7.15 (t, j=8.0 Hz, 2Hz, J, 7.42 (s, 1H). ^{13}C-APT-NMR (400 MHz, $CDCl_3$, TMS, ppm): 55.47 C^1, 164.18 C^2(ipso), 114.43 C^3, 131.17 C^4, 126.34 C^5, 141.84 $C^{5,}$ 107.54 C^7, 118.1 C^8, 131.02 C^9, 127.56 C^{10}, 115.96 C^{11}, 161.70 C^{12}(ipso).

1.3. Synthesis of Compound 2c

4-methoxybenzaldehyde (3.67 mmol, 0.50 g) and 2-(3,5-bis(trifluoromethyl)phenyl)-3-(4-methoxyphenyl)acetonitrile (3.67 mmol, 0.68 g) were reacted. The pale yellow solid substance was obtained as the product (not crystal). Yield: 0.86 g. 77 *%. Anal. Calc.* for $C_{18}H_{11}F_6NO$ (MW= 371.28 g.moL^{-1}): C, 58.23; H, 2.99; N, 3.77. Found: C, 58.15; H, 2.95; N, 3.72. %. FT-IR (KBr, *cm*$^{-1}$) 3071 and 3024$v_{C-H(Ar.)}$, 2210$v_{C≡N}$, 1522, 1576 and 1613$v_{C=C}$ *and* 1121v_{C-F}. ^{1}H-NMR (400 *MHz*, , $CDCl_3$, TMS, ppm): δ= 3.93 (3H, s; $-OCH_3$), 7.04 (2H, *j* = 7.2 Hz, d; Ar-H), 7.61 (1H, s; Ar-CH=C-CN), 7.90 (1H, s; Ar-H), 7.98 (2H, *j* = 7.2 Hz, d; Ar-H), 8.10 (2H, s; Ar-H). ^{13}C-APT-NMR (400 *MHz*, $CDCl_3$, TMS, ppm): δ= 55.57 C^1, 162.43 C^2, 114.69 C^3, 131.90 C^4, 125.49 C^5, 144.94 C^6, 105.50 C^7, 117.59 C^8, 137.23 C^9, 125.70 C^{10}, 124.38 C^{11}, 122.18 C^{12}, 132.44 C^{13}.

2. X-ray Crystallography

Anisotropic thermal ellipsoids were assigned to all atoms except hydrogen atoms, whose positions were refined using the riding model. Comprehensive crystallographic details of the analyzed molecules are summarized in ***Table S3***.

Table S3. Crystallographic results for the compound **2a** and **2b**.

Name	2a	2b
Empirical formula	$C_{16}H_{13}NO$	$C_{16}H_{12}FNO$

Formula weight	235.288	253.27
Temperature (K)	293(2)	150.15
Crystal system	orthorhombic	monoclinic
Space group	$Pna2_1$	$P2_1/n$
Unit cell dimensions		
a (Å)	11.5850(9)	20.501(6)
b (Å)	14.8718(1)	6.673(3)
c (Å)	14.773(2)	20.588(7)
α (°)	90	90
β (°)	90	116.28(4)
γ (°)	90	90
Volume/(Å^3)	2545.2(5)	2525.1(8)
Z	8	8
D_{calc} (g/cm^{-3})	1.228	1.332
Absorption coefficient (mm^{-1})	0.077	0.093
F (000)	992.6	1056.0
Crystal size (mm)	0.59× 0.51× 0.27	0.41 × 0.27 × 0.17
h ranges	-13→9	-24→22
k range	-8→18	-4→7
l range	-10→18	-24→20
Reflections collected/unique	5760/3473	8509/4424
Data / restrains / parameters	3473/1/327	4424/0/346
Goodness of fit on F^2	1.033	0.730
Final *R* indices [$I > 2\sigma(I)$]	R_1 = 0.0537<br>wR_2 = 0.0975	R_1 = 0.0793<br>wR_2 = 0.1009
R indices (all data)	R_1 = 0.1160<br>wR_2 = 0.1273	R_1 = 0.3827<br>wR_2 = 0.1940
Largest difference peak and hole (e Å^{-3})	0.22/-0.20	0.17/-0.26
CCDC #	2431783	2426422

3. Microorganisms and Antimicrobial Activity

The numbers of strain and sources of the obtained microorganisms are presented in Table S4.

Table S4. Bacterial and fungal strains used for antimicrobial activity test.

**Bacterial Strains**	**Fungal Strains**
Bacillus subtilis (ATCC-19659)	*Candida albicans* (ATCC-10231)

Bacillus cereus (ATCC-10876)

Enterococcus faecalis (ATCC 29212)

Escherichia coli (ATCC 25922)

Pseudomonas aeruginosa (ATCC 27853)

Salmonella typhimurium (NRRL-B-4420)

Staphylococcus aureus (ATCC 29213)

Micrococcus luteus (NRRL-B-1018)

Klebsiella pneumoniae (ATCC-70063)

ATCC: American Type Culture Collection; NRRL: Northern Regional Research Laboratory

The compounds **2(a-c)** obtained could only be completely solubilised with dimethyl sulphoxide (DMSO) and the highest possible stock solutions of 50 mg.mL^{-1} were prepared. To these wells 50 µl of the previously prepared substances were transferred using sterile micropipette tips. For comparison, Ampicillin and kanamycin antibiotics for bacteria and Amphotericin B antibiotics for fungus were used as positive controls and DMSO was used as negative control. The prepared petri plates were kept at +4°C for about 1 hour and then the bacterial cultures were incubated at 37°C for 18-24 hours and mould cultures at 27-30°C for 48-72 hours. The zones formed were measured millimetrically with callipers. The experiments were performed with three repetitions.

3.1. Determination of Minimum Inhibition Concentration (MIC)

The minimum inhibition concentration (MIC) values of the compounds **2(a-c)** against the test bacteria used in the study were determined according to the broth microdilution method.[1-2] MHB or SDB was used as test medium and bacterial and yeast suspensions were prepared at 0.5 Mc Farland concentration for each test bacteria. Double layer serial dilutions were prepared in "U" type 96-well microtiter plates. Medium+bacteria suspensions containing only medium in the first row, medium+bacteria in the second row and the final concentrations of the synthesised substances in the other rows between 25 mg.mL^{-1} and 0.097 µg.mL^{-1} were added to the wells with a final volume of 200 µL. Ampicillin, Kanamycin and Amphotericin B standard antibiotics were used as positive controls. Bacteria were incubated at 37±0.1 °C and C. albicans at 30±0.1 °C for 24-48 hours and the lowest concentration without growth was determined as MIC. Tests were performed in two parallel runs and mean values were given as a result. In order to estimate “the minimum bactericidal/fungicidal concentrations (MBC/MFC) of the synthesised acrylonitrile derivatives “needed to indicate 99.5% killing of the original inoculum,” 10 µl was taken from the wells with no observed growth and further sub-cultured onto their agar media (MHA and SDA for bacterial and fungal strains, respectively).[3]

3.2. Cell Lines and Compounds Preparation

The compounds **2a**, **2b**, and **2c** were dissolved in 99.8% Dimethyl sulfoxide (DMSO) respectively, as 10 mM main stock. To prepare the doses of acrylonitrile compounds used in the experiment to be applied to the cells, five different concentrations of 31.25; 62.5; 125; 250 and 500 μM were prepared by diluting the main stock solutions (10 mM) with the medium selected according to the cells. 5-Fluorouracil (5-FU) was selected as a positive control in the study. After the 5-FU main stock solution was dissolved with DMSO to be 10 mM, the concentrations to be used were diluted with the selected media and made ready for the experimental plan. The concentrations used for 5-FU were prepared in the same doses used for acrylonitrile derivatives. For the negative control group, groups containing only media and no substance were used. Prepared acrylonitrile compounds and 5-FU concentrations were added to the wells in three replicates for each dose, and their effects on cell viability at 24 and 48 hours were determined.

Cell Viabilty Assay

Trypan blue solution (Gibco, 1525061) was used to distinguish between living and dead cells in the study. Trypan blue passes uncontrollably through the membrane of dead cells that have lost their cell membrane integrity and stains the cell blue. Living cells, on the other hand, are not stained because they do not allow the dye to pass through. Live cell counts were performed using trypan blue using a cell counter (Logos Luna II).

In order to detach the cells adhering to the flasks from the bottom, 500 μL of 0.25% Trypsin-EDTA (Gibco, 15400054) solution was added and treated for five minutes. When the cells were observed to detach from the bottom under an inverted microscope (Zeiss Primovert, Germany), medium containing 10% FBS was added. After centrifugation at 300 g for 5 minutes and discarding the medium, 1 mL of medium was added and the cells were seeded into 96-well culture dishes as $1x10^4$ cells in 100 μL per well.

Cell Counting Kit 8 (CCK8; Medchem, 146436) was used to determine cell viability. After adding 20 μL of CCK8 solution to each well in the dark, 96-well culture dishes were wrapped with aluminum foil and kept in a CO_2 incubator at 37 ˚C for 2 hr. Then, it was automatically read with a microplate reader (Chromate) at 450 and 630 nm wavelengths. The following formula was used to determine cell viability.

% Viability = (Sample Well−Blank) (Negative Control−Blank)×100 (1)

The half-maximal inhibitory concentration (IC_{50}) was calculated as 50% cell death causing dose compared to the control group. All data are given as the mean percent fraction of control ± SEM. Statistical analysis was done by one-way analysis of variance (ANOVA), followed by Tukey's multiple comparison tests. IBM SPSS Statistics 22 was used to utilize statistical analysis. A p value of less than 0.05 was significant.

4. Optimization and In Silico Studies

4.1. Optimization of the Compounds

Firstly, dihedral angle scan of the title molecules to be optimized were performed, and then the molecular structures were optimized by the DFT method (B3LYP/6-31 G(d,p)) (Becke's three-parameter hybrid model using the Lee-Yang-Parr correlation functional).[4] The optimized molecular structures are shown in Figure S10.

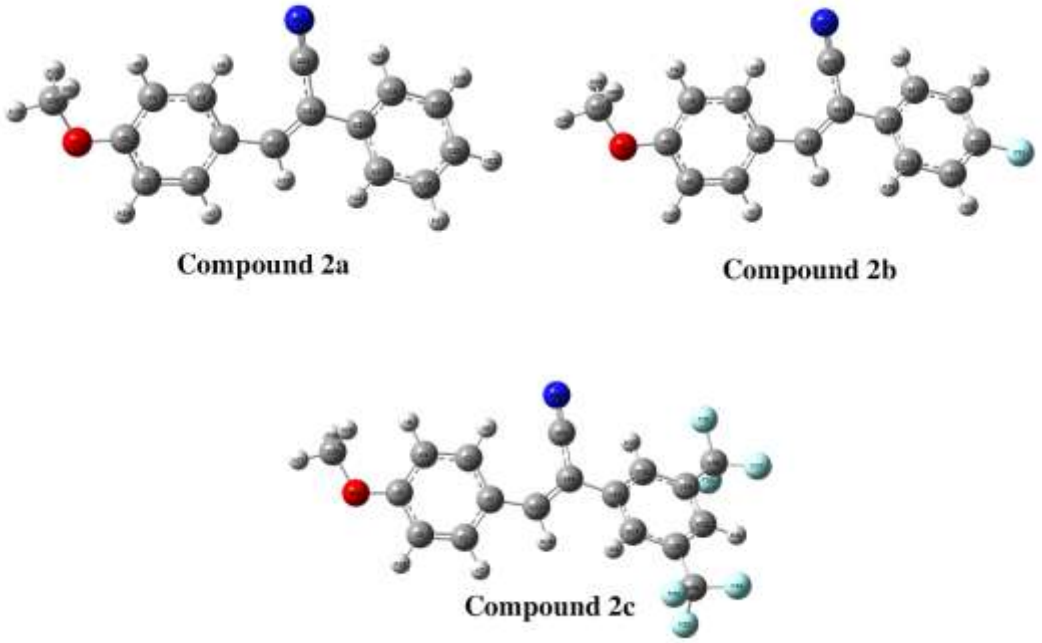

Figure S10. Optimized structures of synthesized compounds.

4.2. Summary of Calculated Data

Table S5. Calculated data including electronic energies and thermal corrections for optimized structures

Calculated Data (a.u.)	2a	2b	2c
Dipole Moment	1.2318	1.5134	2.2384
Polarizability	203.1183	204.4393	231.6357
Electronic Energy	-747.4945	-846.7259	-1421.5634
Zero-point Energy	-747.2477	-846.4874	-1421.3074
Thermal Energy	-747.2322	-846.4711	-1421.2845
Thermal Enthalpy	-747.2313	-846.4701	-1421.2836
Thermal Free Energy	-747.2921	-846.5332	-1421.3642

4.3. XYZ Coordinates of Molecular Geometries

The XYZ coordinates of the theoretically optimized molecular geometries (compounds **2a**, **2b**, and **2c**) are listed in ***Tables S6–S8***.

Table S6. Coordinates of the compound **2a**

Center Number	Atomic Number	Atomic Type	Coordinates (Angstroms)		
			X	Y	Z
1	6	0	-1.803093	-1.463693	-0.809116
2	6	0	-1.051262	-0.564527	-0.018743
3	6	0	-1.718333	0.420117	0.725164
4	6	0	-3.110939	0.528759	0.687676
5	6	0	-3.830760	-0.364245	-0.114975
6	6	0	-3.184829	-1.370014	-0.869277
7	1	0	-1.289680	-2.233768	-1.386778
8	1	0	-1.149965	1.110325	1.351951
9	1	0	-3.607009	1.298739	1.269665
10	1	0	-3.780800	-2.045385	-1.479560
11	8	0	-5.196473	-0.386978	-0.264111
12	6	0	0.406109	-0.708537	0.015678
13	1	0	0.760485	-1.747577	0.063813
14	6	0	1.311061	0.290840	-0.025619
15	6	0	2.770374	0.029082	0.024599
16	6	0	3.613726	0.552311	-0.968632
17	6	0	3.308768	-0.746080	1.063603
18	6	0	4.985530	0.293038	-0.923278
19	1	0	3.198696	1.158818	-1.773394
20	6	0	4.682592	-0.999003	1.102691
21	1	0	2.659572	-1.141732	1.842915
22	6	0	5.521696	-0.482421	0.110383
23	1	0	6.591535	-0.681115	0.143394
24	6	0	0.949835	1.673924	-0.140494
25	7	0	0.675670	2.793801	-0.234940
26	1	0	5.638898	0.698208	-1.695088
27	1	0	5.100019	-1.598545	1.910470
28	6	0	-5.973060	0.616378	0.435973
29	1	0	-5.693160	1.618391	0.097740
30	1	0	-6.995111	0.366560	0.121449
31	1	0	-5.855866	0.500725	1.517089

Table S7. Coordinates of the compound **2b**

Center Number	Atomic Number	Atomic Type	Coordinates (Angstroms)		
			X	Y	Z
1	6	0	-2.188954	-1.464005	-0.814202
2	6	0	-1.451300	-0.550312	-0.026716
3	6	0	-2.134631	0.419675	0.721918
4	6	0	-3.529155	0.500058	0.691537
5	6	0	-4.235053	-0.406360	-0.108739
6	6	0	-3.572360	-1.398173	-0.867410
7	1	0	-1.663162	-2.223232	-1.395185
8	1	0	-1.577650	1.120269	1.347412
9	1	0	-4.037975	1.259020	1.277174
10	1	0	-4.157737	-2.084972	-1.475435
11	8	0	-5.600113	-0.456312	-0.251454
12	6	0	0.008448	-0.664969	-0.000875
13	1	0	0.383890	-1.697105	0.036004
14	6	0	0.892951	0.352973	-0.037686
15	6	0	2.355979	0.120722	0.006088
16	6	0	2.914484	-0.627270	1.056145
17	6	0	3.180723	0.646550	-1.003889
18	6	0	4.290307	-0.861720	1.102161
19	1	0	2.273193	-1.021778	1.845133
20	6	0	4.557920	0.422514	-0.977848
21	1	0	2.742755	1.232598	-1.814123
22	6	0	5.080585	-0.330011	0.078918
23	6	0	0.505392	1.729922	-0.140589
24	7	0	0.210520	2.845133	-0.226253
25	1	0	4.730391	-1.439078	1.914253
26	1	0	5.202906	0.825779	-1.757984
27	6	0	-6.394212	0.528570	0.455587
28	1	0	-6.138079	1.537083	0.117860
29	1	0	-7.412417	0.257162	0.146209
30	1	0	-6.268484	0.413056	1.535752
31	9	0	6.395877	-0.550359	0.112438

Table S8. Coordinates of the compound **2c**

Center Number	Atomic Number	Atomic Type	Coordinates (Angstroms)		
			X	Y	Z
1	6	0	3.737957	1.187050	-1.113620
2	6	0	3.061490	0.268687	-0.273870
3	6	0	3.849587	-0.530844	0.574570
4	6	0	5.236496	-0.434601	0.572136
5	6	0	5.882018	0.469664	-0.282159
6	6	0	5.113967	1.282063	-1.133106
7	1	0	3.154573	1.826665	-1.769999
8	1	0	3.381631	-1.232577	1.250378
9	1	0	5.805429	-1.066316	1.241432
10	1	0	5.628144	1.977638	-1.785207
11	8	0	7.227720	0.657604	-0.359629
12	6	0	1.603838	0.282673	-0.331217
13	1	0	1.216141	1.105632	-0.923524
14	6	0	0.651163	-0.541674	0.189998
15	6	0	-0.799503	-0.305077	0.053381
16	6	0	-1.722380	-1.316505	0.365251
17	6	0	-1.303148	0.948500	-0.330772
18	6	0	-3.092470	-1.077195	0.306610
19	1	0	-1.375350	-2.286950	0.694528
20	6	0	-2.670184	1.175937	-0.405388
21	1	0	-0.639906	1.773878	-0.548747
22	6	0	-3.580748	0.170244	-0.075386
23	6	0	1.045365	-1.713196	0.927640
24	7	0	1.318625	-2.677898	1.523211
25	6	0	8.056533	-0.141445	0.508285
26	1	0	7.919314	-1.201960	0.292638
27	1	0	9.071998	0.160881	0.270637
28	1	0	7.836400	0.074968	1.554388
29	6	0	-3.223980	2.497439	-0.865124
30	6	0	-4.096082	-2.148693	0.645095
31	1	0	-4.644154	0.378495	-0.079169
32	9	0	-2.275426	3.456647	-0.932987
33	9	0	-3.794779	2.398750	-2.089265
34	9	0	-4.193991	2.935466	-0.032037
35	9	0	-3.524719	-3.268381	1.133644
36	9	0	-4.836348	-2.503722	-0.431555
37	9	0	-4.966366	-1.690662	1.578407

4.4. In Silico Studies

Before performing the molecular docking, all water molecules were removed, polar hydrogen atoms were added, and then charged with Total Kollman Charges. The Auto Grid map was utilized to set the proper size of the grid box. AutoDockTools 1.5.7 [5] was employed to determine the proper size of the grid box for the potential binding site. The predetermined sizes of the grid for docking with 6O0K, 6GU6, 2AZ5, and 1MWT are given in ***Table S9.*** Lamarckian genetic algorithm [6] embedded in AutoDock was used to conduct the docking calculations. In addition, Discovery Studio Visualizer v21.1.0.20298 software [7] was used to visualise the docked active sites and H-bond interactions between ligands and receptors.

Table S9. Grid options of the protein site for molecular docking.

Grid Options / PDB	6O0K	6GU6	2AZ5	1MWT
x-dimension	26	20	20	20
y-dimension	26	20	20	20
z-dimension	26	20	20	20
Spacing (angstrom)	0.375	0.375	0.375	0.375
x-center	-15.320	23.640	-19.400	27.990
y-center	2.210	21.820	74.650	28.940
z-center	-9.590	-1.900	33.840	87.530

5. Details of Single Crystal X-ray Crystallography

The single-crystal X-ray structure of molecules **2a** and **2b**, whose ORTEP views are provided in ***Figure S11***, reveals an arrangement featuring π-conjugated aromatic benzene rings that contribute to an extended π-system, along with methoxy and nitrile functional groups. Unfortunately, the single crystal of **2c** could not be obtained. The molecule **2b** consist of the flouride hetereoatom in the terminal side of molecule backbone. The overall molecular conformation is non-planar, with distinct torsional angles between the two ring systems of 58.7 for **2a**, 38.6 for **2b**, indicating steric effects and electronic influences governing the three-dimensional structure. The bond lengths in both molecular structures (C-N:1.13Å and 1.15Å; C-O:1.43Å; (C-C) *average*:1.39Å) are consistent with the values reported in the literatüre.[8-13] In addition, the asymmetric unit of the molecules contains two independent molecules, likely due to intermolecular interactions, conformational flexibility, or symmetry constraints. These molecules exhibit slight geometric differences influenced by hydrogen bonding or steric effects, contributing to crystal stabilization. Despite these variations, the bond lengths and angles remain within expected literature values. This structural arrangement highlights the role of intermolecular forces in determining the crystal packing and material properties.

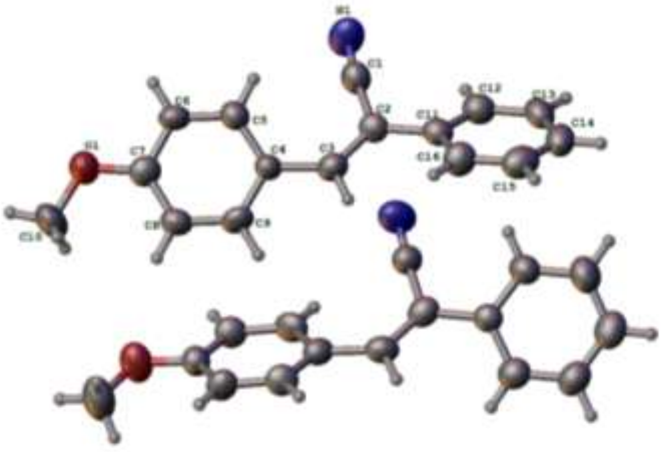

2a

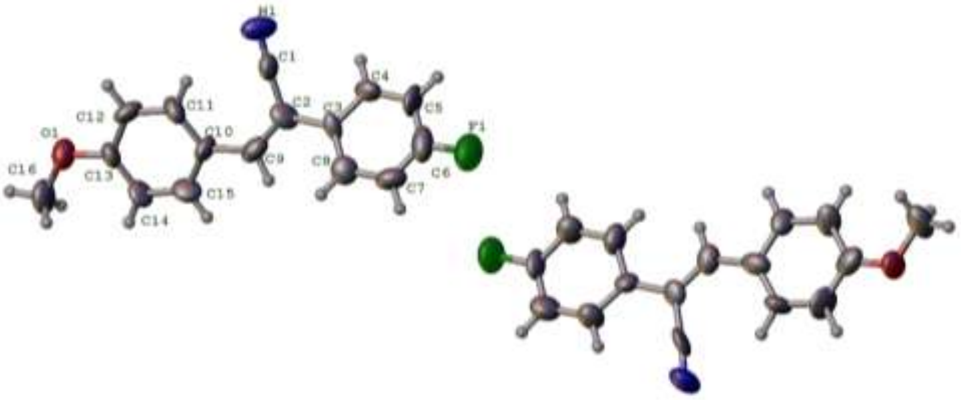

2b

Figure S11. The ORTEP views of the compounds 2a and 2b.

The packing structure of the crystal **2b** is stabilized through strong nonclassical hydrogen bonding (C-H…N, C-H…F) along the (001) plane in the layered network. The dimer formation plays a crucial role in stabilizing the overall packing. The dimers are primarily held together by C-H…N hydrogen bonds that facilitate a head-to-head or face-to-face alignment of the molecules. C-H..F interactions further support the dimer structure by providing additional stabilization (***Figure S12***). Their periodic arrangement contributes to the long-range order and structural stability of the crystal.[14]

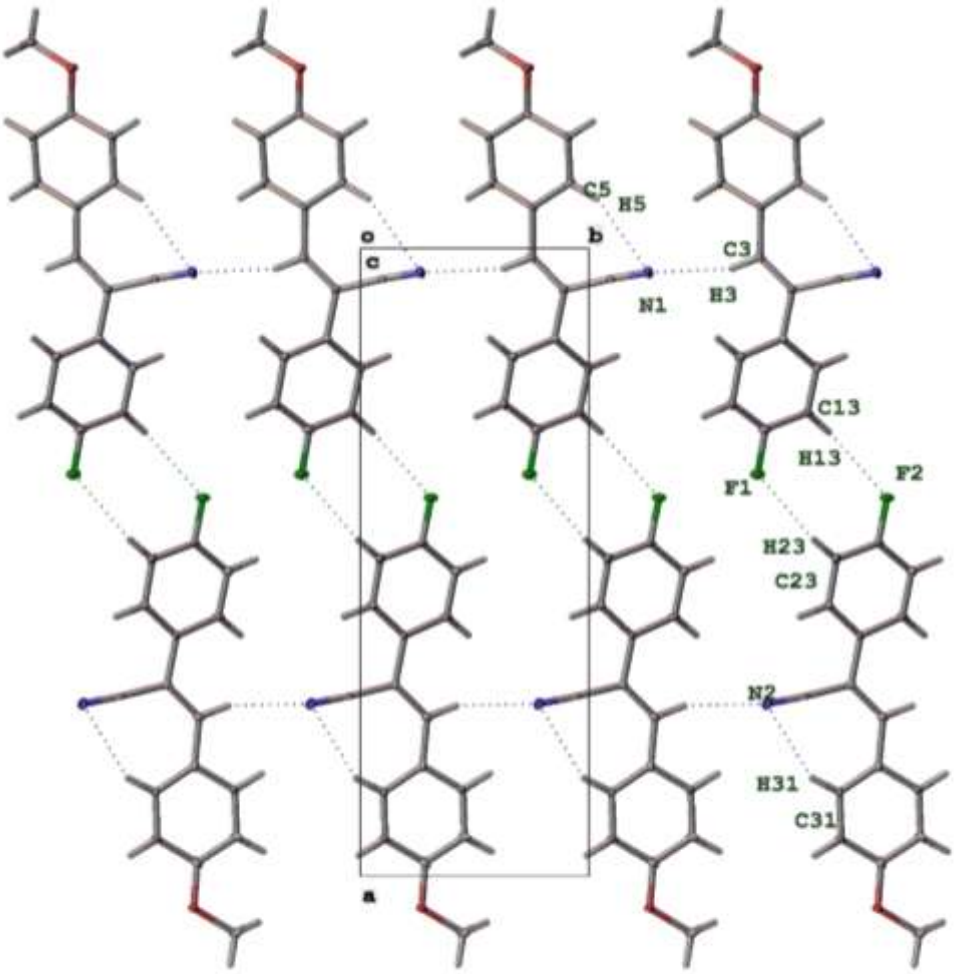

***Figure S12*.** The molecular arrangement of the **2b** along the (001) plane with the dimeric motifs.

Furthermore, π-π stacking interactions between aromatic rings may also play a role in reinforcing the dimeric units. The planar nature of the molecules allows for efficient stacking, which enhances the compactness and rigidity of the crystal. The π-π stacking interactions occur between aromatic rings, with distances measured 4.858 Å, indicating strong face-to-face contacts between the planar aromatic systems. The combination of hydrogen bonding, fluorine interactions, and possible π-π stacking leads to a well-ordered and stable crystal packing arrangement (***Figure S13***).

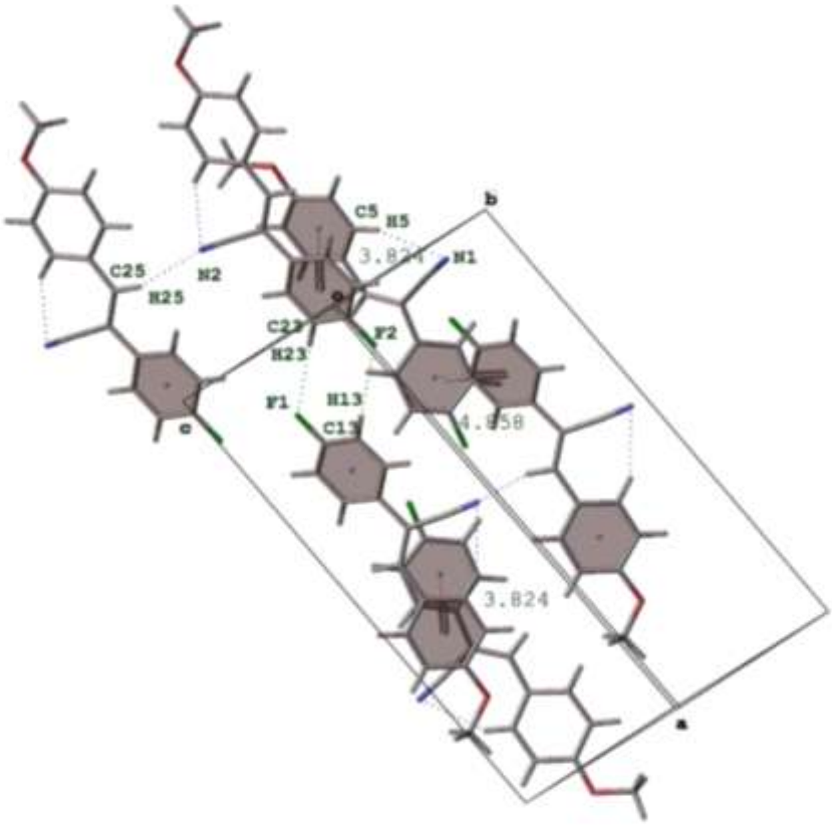

Figure S13. The 2D molecular arrangement of the **2b** along the (011) plane with the stacking interactions**.**

C-H…π interaction in the crystal structure of **2b** plays a complementary role alongside hydrogen bonding and π-π stacking, helping to maintain three-dimensional structural stability. The measured interaction distance of 2.760 Å falls within the typical range for effective C-H…π interactions (2.5–3.0 Å), indicating a strong non-covalent interaction (***Figure S14***).

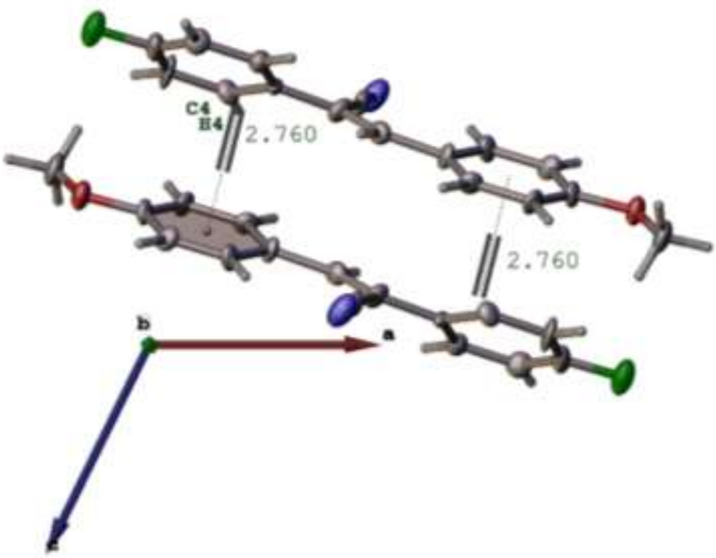

Figure S14. The 2D dimeric arrangement of the **2b** along the (101) plane with the C-H…π interactions.

6. Figures S1-S9

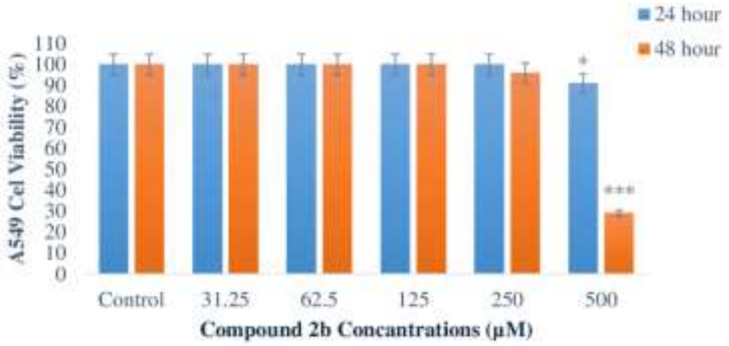

Figure S1. Cytotoxic effects of compound **2b** on A549 cells after 24 and 48 hours. The control group was accepted as 100% (*****: p<0.05; *******: p<0.001)

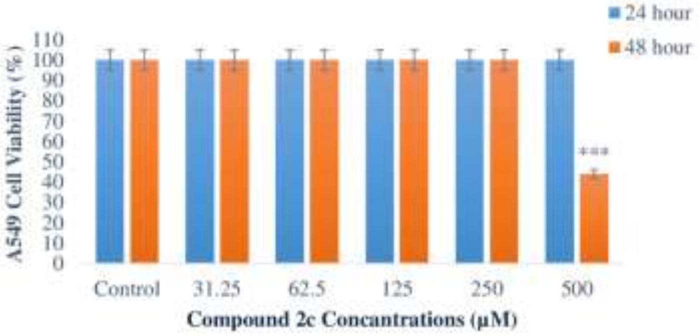

Figure S2. Cytotoxic effects of compound **2c** on A549 cells after 24 and 48 hours. The control group was accepted as 100% (*******: p<0.001)

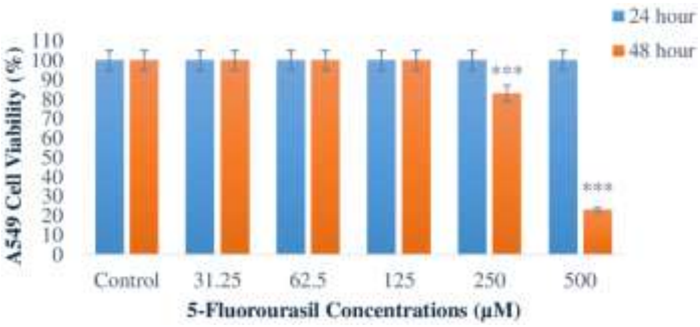

Figure S3. Cytotoxic effects of 5-Fluorourasil on A549 cells after 24 and 48 hours. The control group was accepted as 100% (*******: p<0.001)

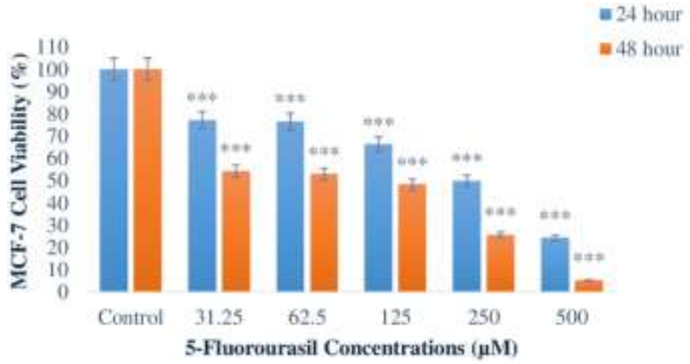

Figure S4. Cytotoxic effects of 5-Fluorourasil on MCF-7 cells after 24 and 48 hours. The control group was accepted as 100% (*******: p<0.001)

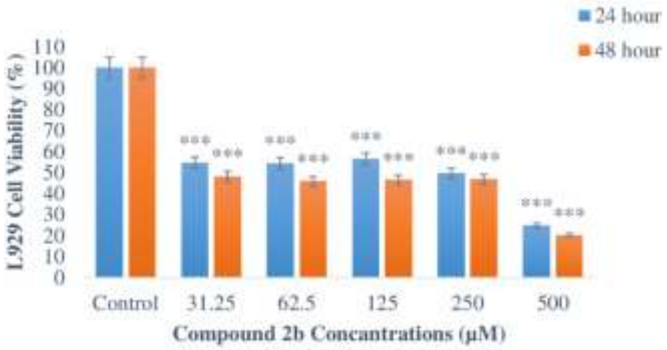

Figure S5. Cytotoxic effects of compound **2b** on L929 cells after 24 and 48 hours. The control group was accepted as 100% (*******: p<0.001)

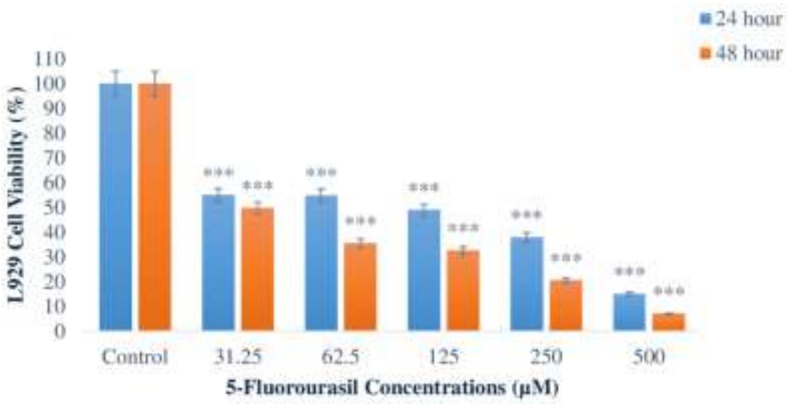

Figure S6. Cytotoxic effects of 5-Fluorourasil on L929 cells after 24 and 48 hours. The control group was accepted as 100% (*****: $p<0.001$)

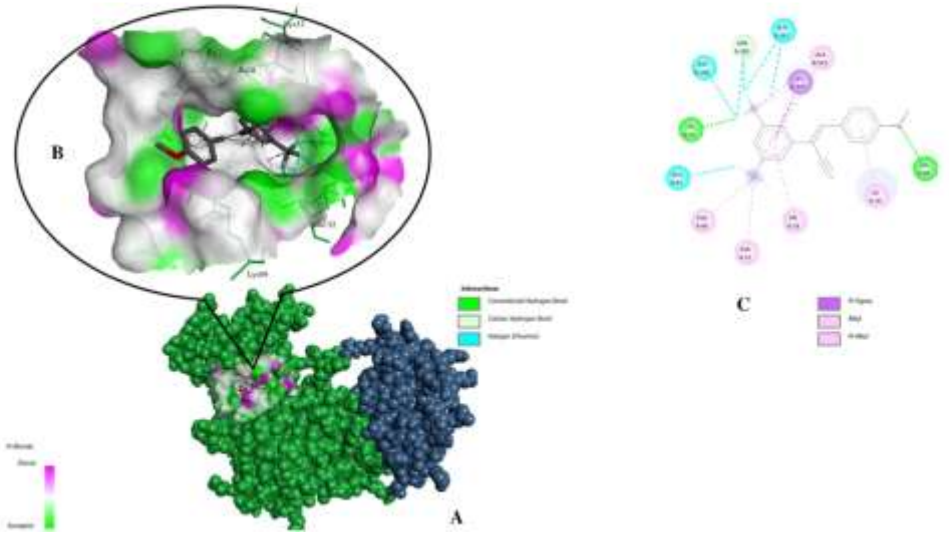

Figure S7. Binding pose profile (A), three-dimensional (B) and two-dimensional (C) representation of 6GU6-ligand **2c** interaction with large binding energy as a result of molecular docking.

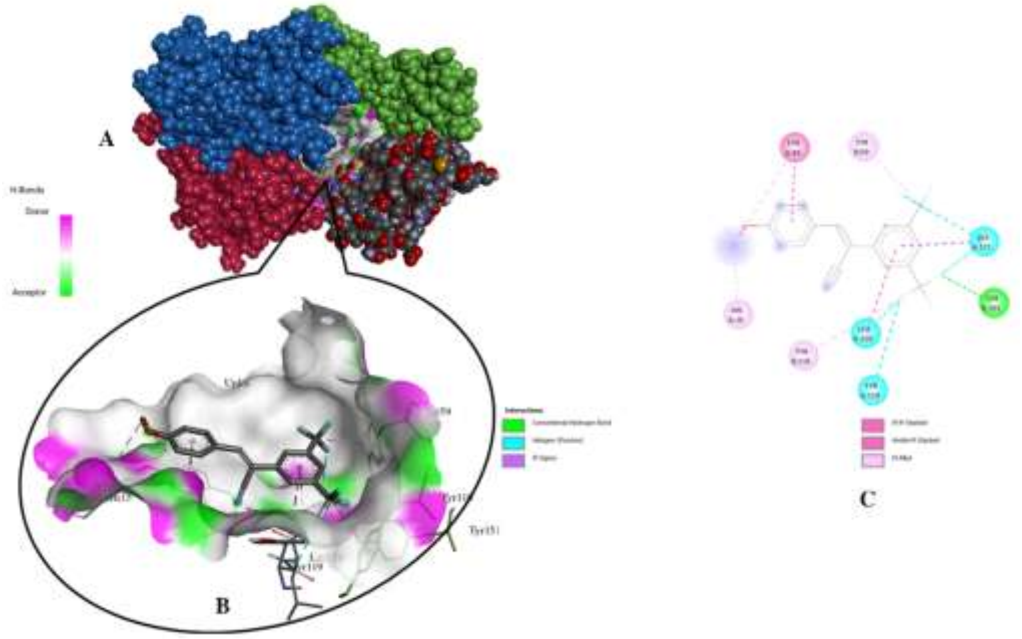

Figure S8. Binding pose profile (A), three-dimensional (B) and two-dimensional (C) representation of 2AZ5-ligand **2c** interaction with large binding energy as a result of molecular docking.

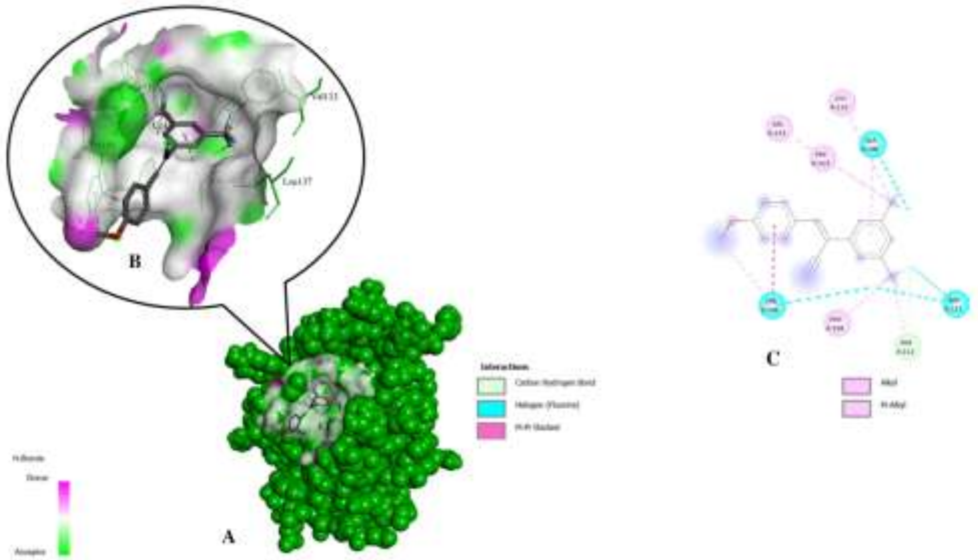

Figure S9. Binding pose profile (A), three-dimensional (B) and two-dimensional (C) representation of 6O0K-ligand **2c** interaction with large binding energy as a result of molecular docking.

7. References

[1] Clinical and Laboratory Standards Institute. M44-A2: Method for Antifungal Disk Diffusion Susceptibility Testing of Yeasts, *Approved Guideline—Second Edition*. **2009**, CLSI. M44-A2, *29* (17).

[2] Clinical and Laboratory Standards Institute. M02-A12: Performance Standards for Antimicrobial Disk Susceptibility Tests, *Approved Standard—Twelfth Edition*. **2015**, CLSI. *35*: 73.

[3] Y. Rukayadi, S. Han, D. Yong, J. K. Hwang, "In vitro antibacterial activity of panduratin A against enterococci clinical isolates" *Biol. Pharm. Bull.* **2010**, *33(9)*, 1489. https://doi.org/10.1248/bpb.33.1489

[4] A. D. Becke, "Density-functional thermochemistry. I. The effect of the exchange-only gradient correction" *J. Chem. Phys.* **1992**, *96*, 2155. https://doi.org/10.1063/1.462066.

[5] M. F. Sanner, "Python: a programming language for software integration and development" *J. Mol. Graph. Model.* **1999**, *17*, 57.

[6] G. M. Morris, D. S. Goodsell, R. S. Halliday, R. Huey, W. E. Hart, R. K. Belew, A. J. Olson, "Automated docking using a Lamarckian genetic algorithm and an empirical binding free energy function" *J. Comput. Chem.* **1998**, *19*, 1639. https://doi.org/10.1002/(SICI)1096-987X(19981115)19:14<1639::AID-JCC10>3.0.CO;2-B.

[7] Dassault Systèmes BIOVIA. Discovery Studio Modeling Environment **2017**, Dassault Systèmes; San Diego, CA, USA.

[8] Y. Zhang, S. Wang, "Acrylonitrile-based polymers: Synthesis, properties, and applications" *Polym. Rev.* **2023**, *63*(2), 345. https://doi.org/10.1080/15583724.2023.1234567

[9] J. Li, H. Chen, X. Liu, "Advances in the functionalization of acrylonitrile derivatives for biomedical applications" *J. Med. Chem.* **2022**, *65*(14), 9876. https://doi.org/10.1021/acs.jmedchem.2c01234

[10] R. Kumar, P. Singh, "Catalytic hydrogenation of acrylonitrile derivatives: A comprehensive review" *ACS Catal.* **2021**, *11*(10), 6255. https://doi.org/10.1021/acscatal.1c01567

[11] M. Garcia, D. Lopez, "Environmental impact of acrylonitrile and its derivatives: A systematic review" *Environ. Sci. Technol.* **2020**, *54*(18), 11234. https://doi.org/10.1021/acs.est.0c04567

[12] T. Nguyen, L. Tran, "Electrochemical behavior of acrylonitrile-based compounds in energy storage systems" *J. Electrochem. Soc.* **2019**, *166*(12), A2575. https://doi.org/10.1149/2.0451912jes

[13] S. Patel, M. Desai, "Synthesis and characterization of novel acrylonitrile-based copolymers for industrial applications" *Ind. Eng. Chem. Res.* **2018**, *57*(22), 7654. https://doi.org/10.1021/acs.iecr.8b01234

[14] L. Wang, Y. Zhao, “Photopolymerization kinetics of acrylonitrile derivatives under UV irradiation“ *Macromol.* **2017**, *50*(15), 5890. https://doi.org/10.1021/acs.macromol.7b01234
