# Supplementary material for: Crystal Structure–Activity Relationship of Some MeO Phenylacrylonitriles: Dual Antimicrobial–Cytotoxic Effects and in Silico Perspectives
Source: ChemistryOpen. 2025 Jun 12;14(11):e202500280. doi: 10.1002/open.202500280 (PMC12598805; doi:10.1002/open.202500280)

1. 2a Compound FT-IR, ^{1}H NMR and ^{13}C-APT NMR Spectra

1.1. FT-IR Spectrum (2a)

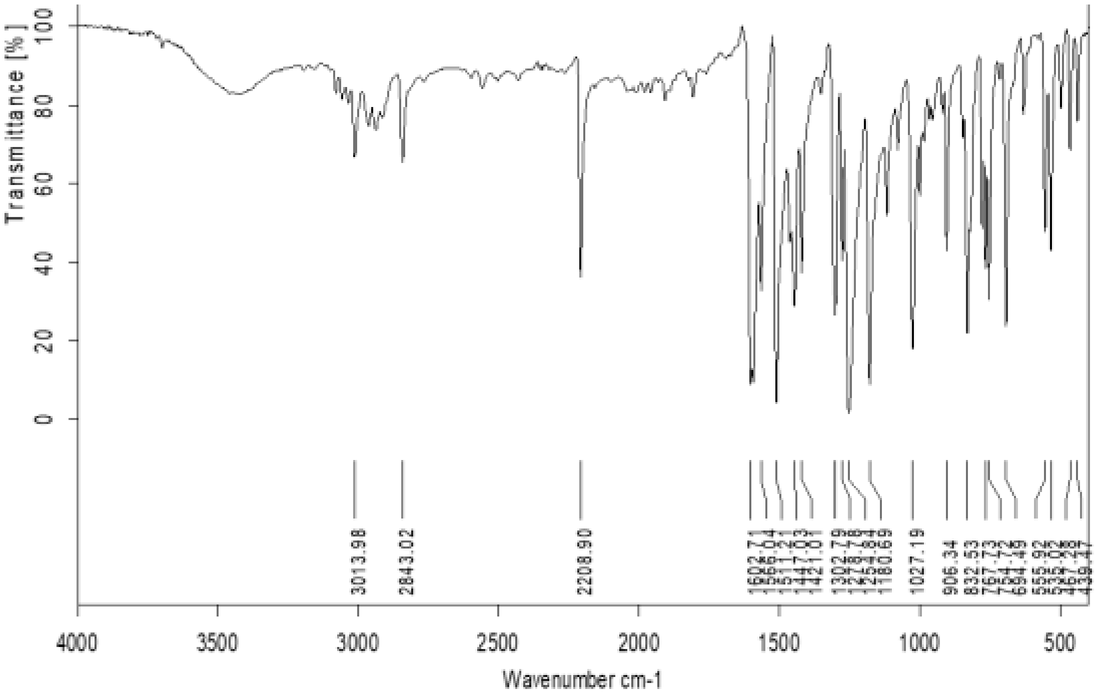

1.2. ^{1}H NMR NMR Spectrum (2a)

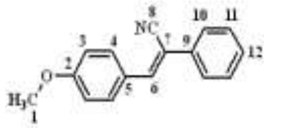

(2a)

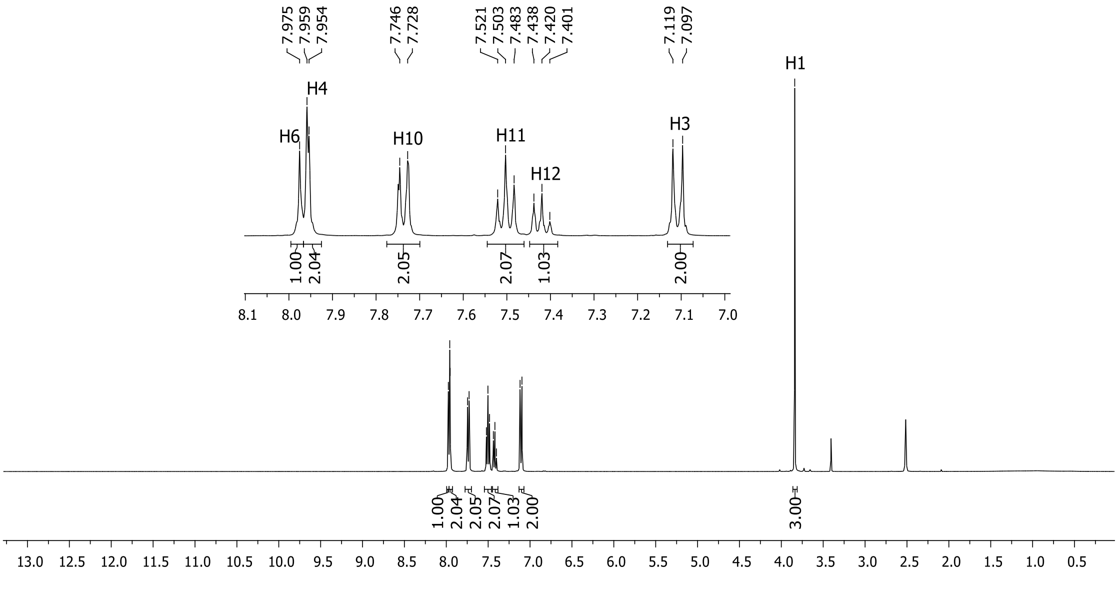

1.3. ^{13}C -APT NMR Spectrum (2a)

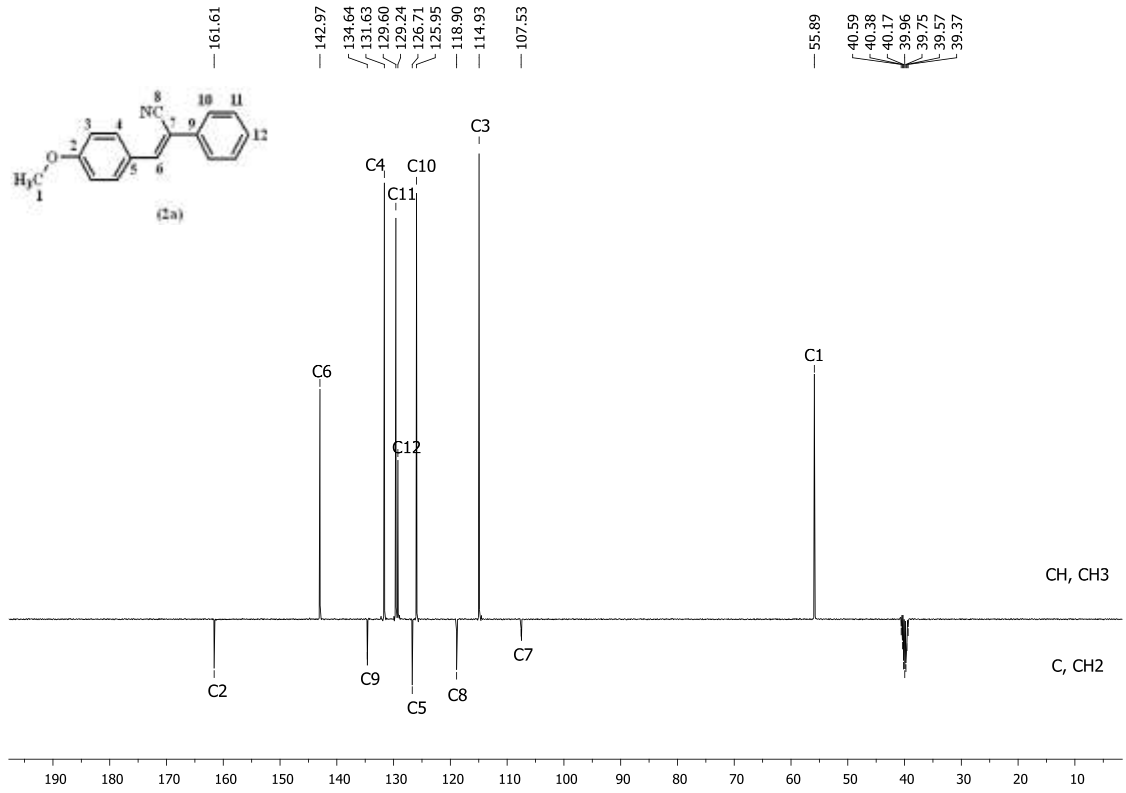

2. 2b Compound FT-IR, ^{1}H NMR and ^{13}C-APT NMR Spectra

2.1. FT-IR Spectrum (2b)

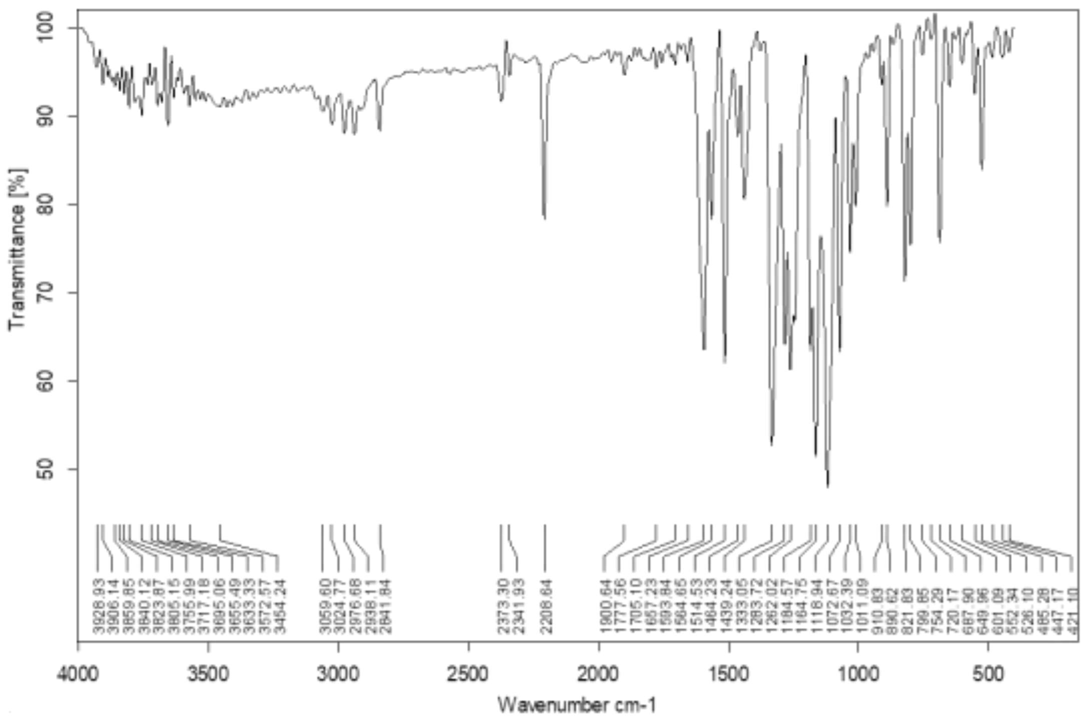

2.2. ^{1}H NMR NMR Spectrum (2b)

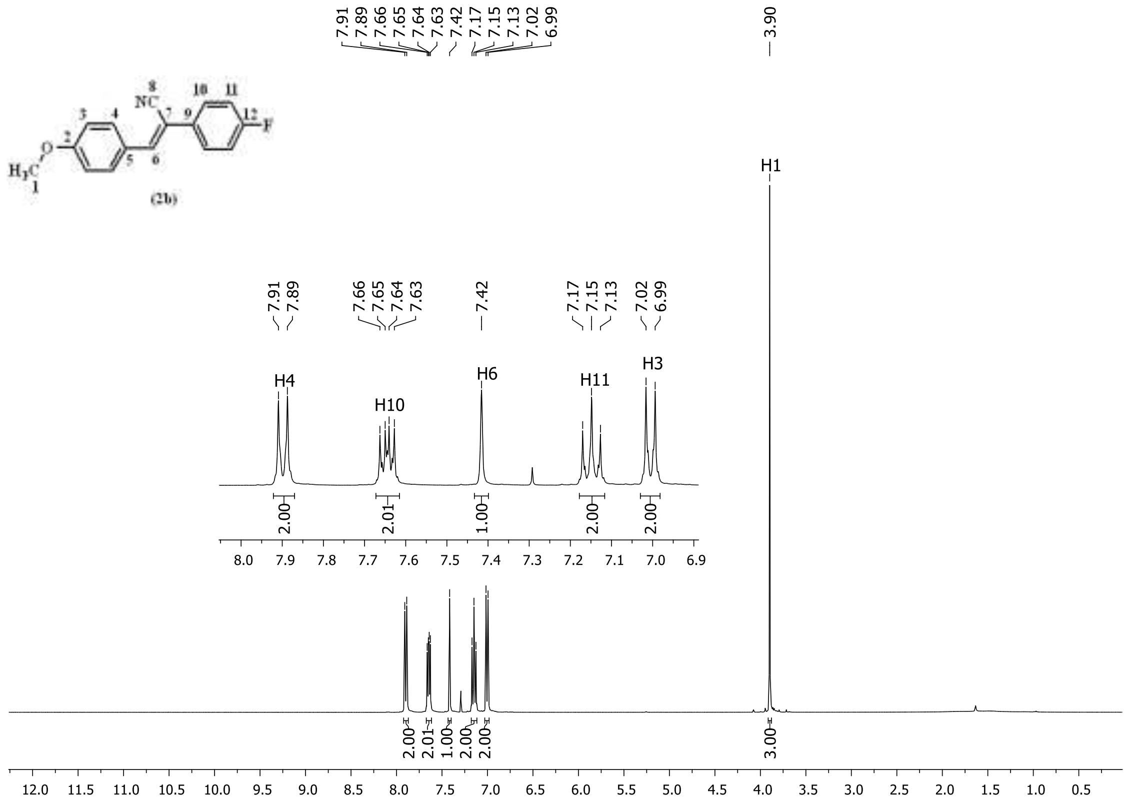

2.3. ^{13}C -APT NMR Spectrum (2b)

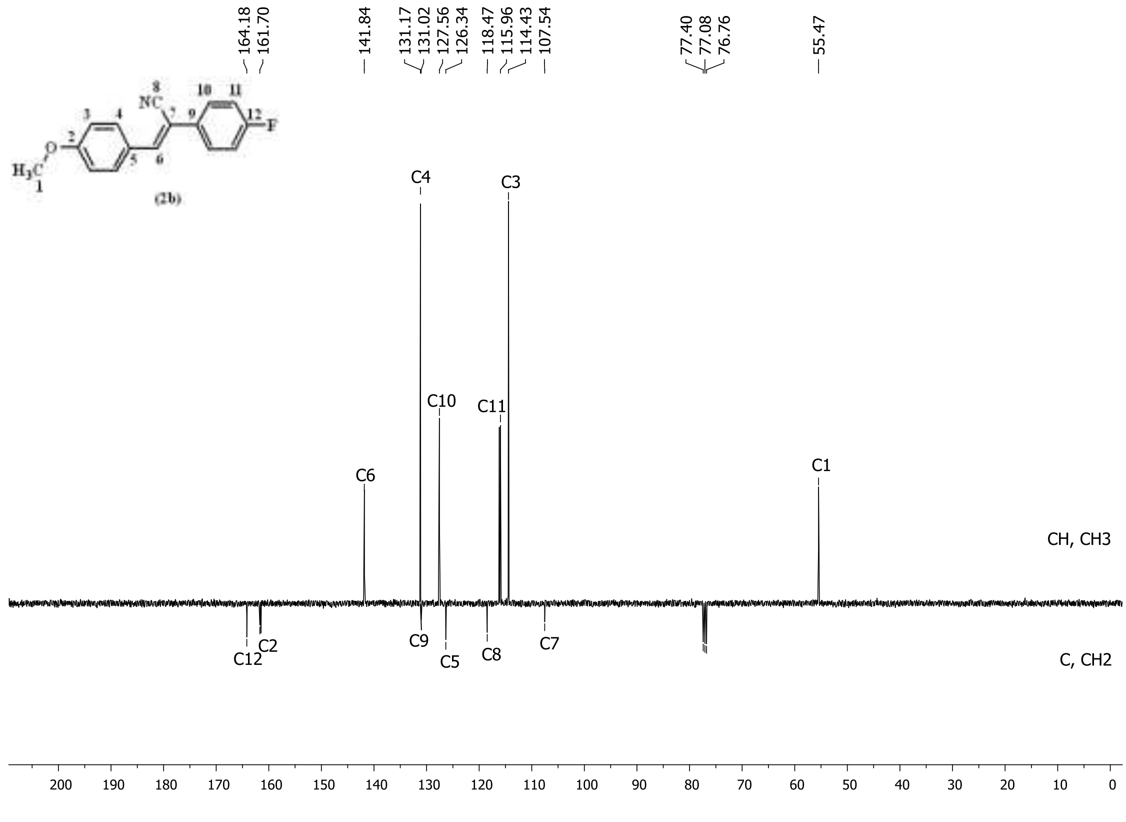

3. 2c Compound FT-IR, ^{1}H NMR and ^{13}C-APT NMR Spectra

3.1. FT-IR Spectrum (2c)

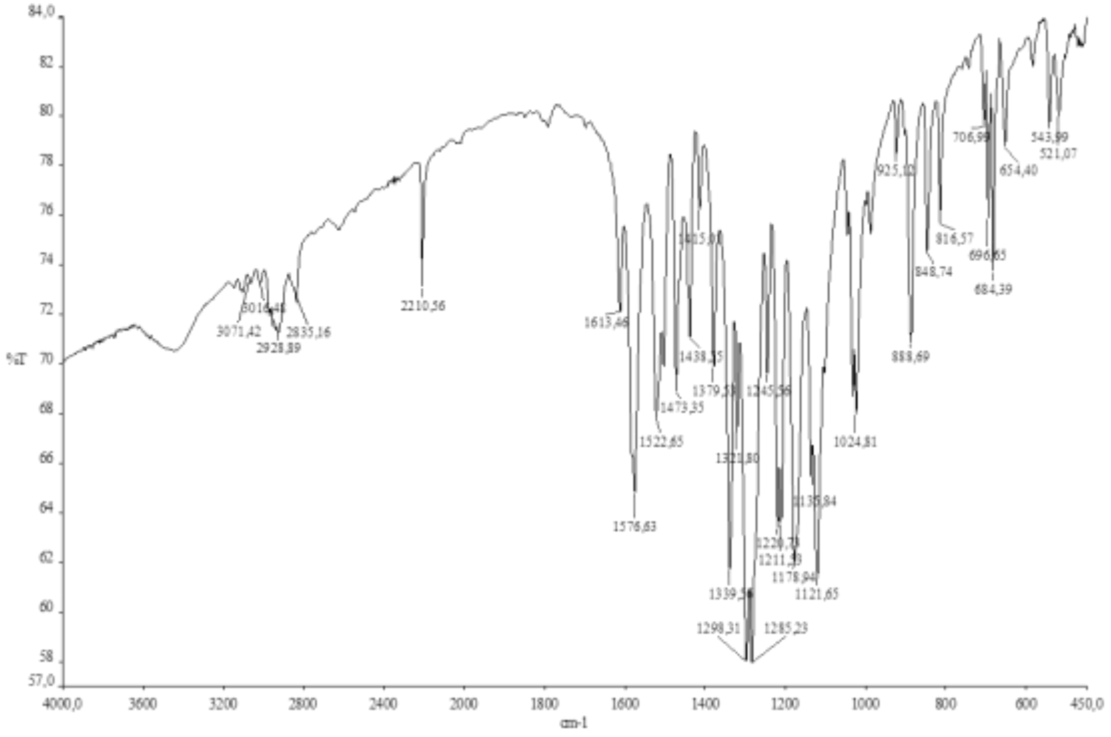

3.2. ^{1}H NMR NMR Spectrum (2c)

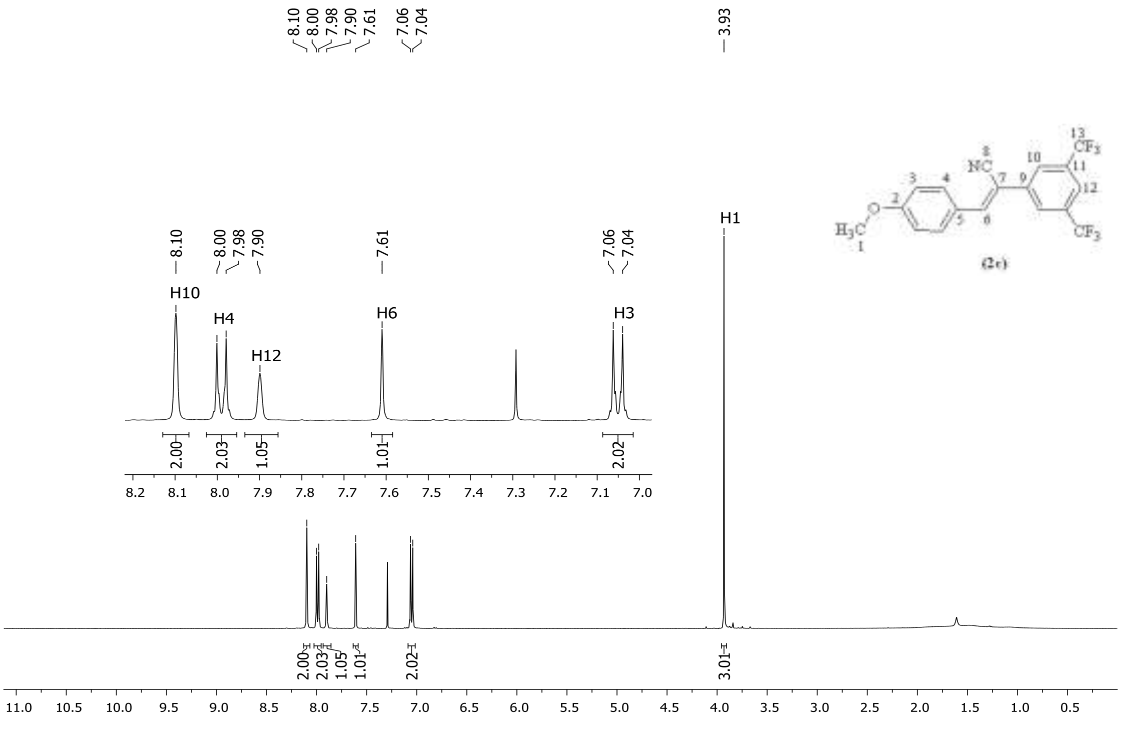

3.3. ^{13}C -APT NMR Spectrum (2c)

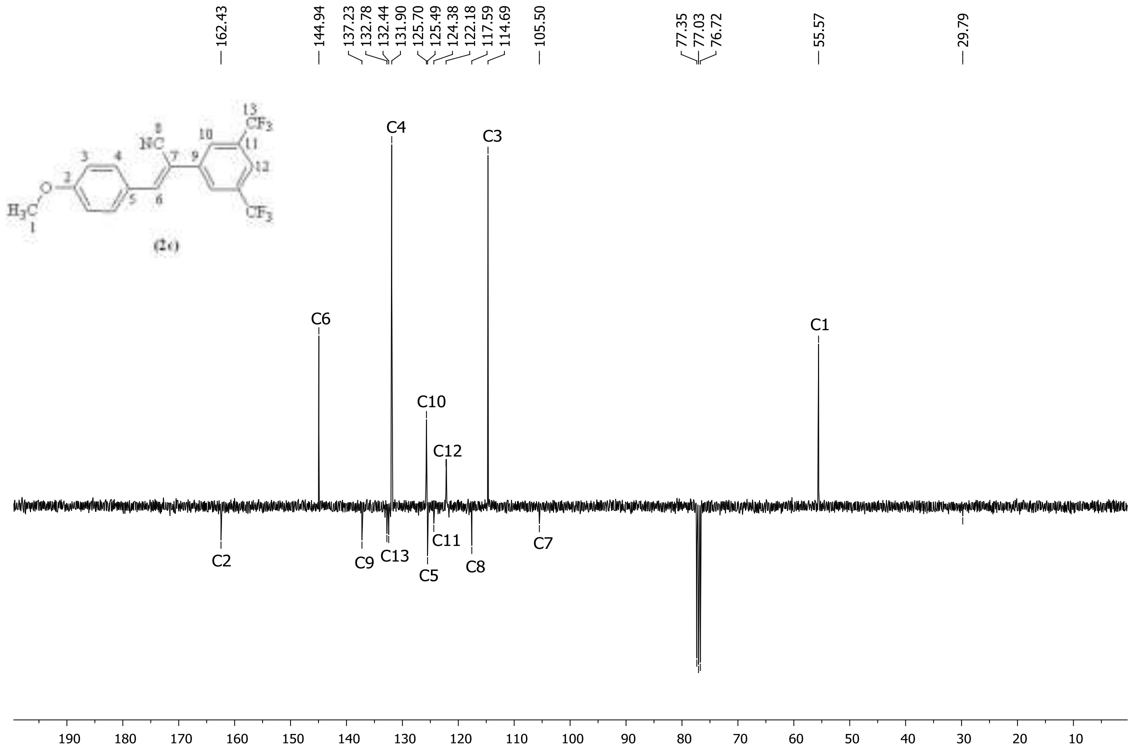

Supplement: Supplementary file 1 — Supplementary Material [file OPEN-14-e202500280-s001.zip › open202500280-sup-0002-suppdata-S2.pdf]
